# Mechanical Energy Drives Dissipative Self-Assembly of Nanocoacervates into Vesicles with Cell-like Properties

**DOI:** 10.1021/jacs.5c14198

**Published:** 2025-12-23

**Authors:** Francesco Vicentini, Aina Rebasa-Vallverdu, Martina Conti, Simone Dal Zilio, Aharon Steffè, Wuge H. Briscoe, Pierangelo Gobbo

**Affiliations:** † Department of Chemical and Pharmaceutical Sciences, 9315 University of Trieste, Via L. Giorgieri 1, Trieste 34127, Italy; ‡ National Interuniversity Consortium of Materials Science and Technology Unit of Trieste, Via G. Giusti 9, Firenze 50121, Italy; § CNR-IOM - Istituto Officina Dei Materiali, 518735 Consiglio Nazionale Delle Ricerche, Area Science Park, Basovizza, Strada Statale 14, Km 163,5, Trieste 34149, Italy; ∥ School of Chemistry, University of Bristol, Cantock’s Close, Bristol BS8 1TS, U.K.

## Abstract

Dissipative self-assembly, which relies on continuous energy input to form and sustain functional structures, underpins the adaptive behaviors of biological systems and is essential for creating synthetic materials with life-like properties. While chemical, thermal, photonic, or electrical energy sources have been used for dissipative self-assembly of nanostructures, this work pioneers mechanical energy as a novel driver to create dissipative polyelectrolyte micrometrical vesicles, with a half-life of ca. 2 days that exhibit cell-like properties such as selective molecular uptake and catalytic functionality. Our strategy works with different polyelectrolyte systems, including DNA and peptides, suggesting relevance to natural systems and the origins of life. Finally, we demonstrate that mechanical energy can also drive the evolution of distinct dissipative vesicle populations into a single, higher-order population with advanced compartmentalization and enhanced synthetic capabilities. Our work establishes mechanical energy as a key driver of dissipative self-assembly, with implications for life-like materials engineering, biotechnology, and microreactor design.

## Graphical abstract

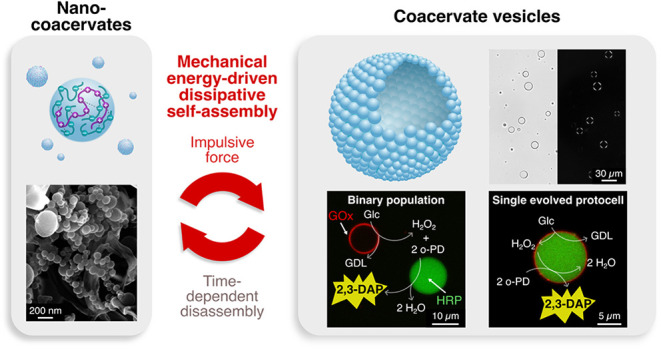

## Introduction

Self-assembly is Nature’s preferred method for building chemical complexity from simple molecular building blocks and constructing biological architectures and functions.^1–3^ Self-assembly processes operate through two distinct thermodynamic pathways.^4^ In the first pathway, they are driven by free energy minimization, creating thermodynamically stable structures at equilibrium. Examples include the formation of lipid bilayers from amphiphilic molecules, crystallization processes, and the folding of proteins and nucleic acids.

In contrast, the second pathway leads to nonequilibrium (or out-of-equilibrium) metastable structures requiring a continuous energy input to maintain their existence and function. When this energy input ceases, the structures dissipate.^5–8^ This more sophisticated form, known as dissipative (or dynamic) self-assembly, enables spatiotemporal control over assembled structures and underpins the adaptive and intelligent behaviors of biological systems.^9,10^ For instance, localized pH shifts or calcium ion concentration increases can trigger the dissipative assembly of actin filaments or microtubules, which are structures responsible for critical cellular processes, including division, movement, and intracellular transport.^11,12^

While equilibrium self-assembly has become a well-established technique in modern science, yielding diverse functional structures and materials, the importance of mastering transient self-assembled structures through dissipative processes has only been recognized recently. The latest advances in this field have demonstrated the potential of dissipative self-assembly to generate a variety of dynamic architectures, including nanoparticle aggregates,^13,14^ fibers,^15,16^ nanocrystals,^17,18^ vesicular nanoreactors,^19,20^ and active droplets.^21–24^ As the field advances, it promises to lead to unprecedented life-like technologies exhibiting adaptability, learning capabilities, sophisticated reactivity, or higher-order properties regulated by external energy sources.^25,26^

In exploring energy forms that can drive dissipative self-assembly, thus far researchers have successfully utilized chemical energy,^27^ light,^28^ electric fields,^29^ and thermal energy.^30^ Despite its simplicity, scalability, and waste-free nature, mechanical energy has remained an underexplored driver of dissipative self-assembly, even though it is widely present in natural systems across the mesoscale, from cellular processes to geological phenomena.^31^ To date, mechanical energy has primarily been employed to assemble or disassemble structures at thermodynamic equilibrium,^32–34^ to oxidize a chemical fuel and trigger dissipative self-assembly,^35,36^ or for the dissipative shear thickening of polymer-based systems.^37,38^ Absent from the literature, however, are examples where mechanical energy alone produces well-defined dissipative self-assembled structures.

Here, we demonstrate that mechanical energy, particularly in the form of impulsive forces, can drive the dissipative self-assembly of microcompartments with cell-like properties, termed *coacervate vesicles*. Coacervate vesicles represent an emergent protocell model that combines the remarkable properties of complex coacervate microdroplets (i.e., their ability to selectively concentrate and protect biomolecules,^39,40^ sustain diverse biochemical reactions,^41,42^ and dynamically evolve^21,43^ ) with the enhanced stability and microcompartmentalization afforded by a vesicular architecture.^44^ While coacervate vesicles have previously been formed only through chemically driven reconfiguration of coacervate *micro*droplets,^45–47^ herein we present a fundamentally new bottom-up approach that utilizes mechanical energy to drive the dissipative self-assembly of coacervate vesicles from nanocoacervate building units (Figure 1). Nanocoacervates (ca. 120 nm in diameter) were prepared by mixing polyanions and polycations at concentrations near but outside the coacervation phase boundary.^48^ Upon application of an impulsive mechanical force via vigorous manual shaking, these nanodroplets spontaneously assembled into coacervate vesicles with a half-life of ca. 2 days.

**Figure 1:**
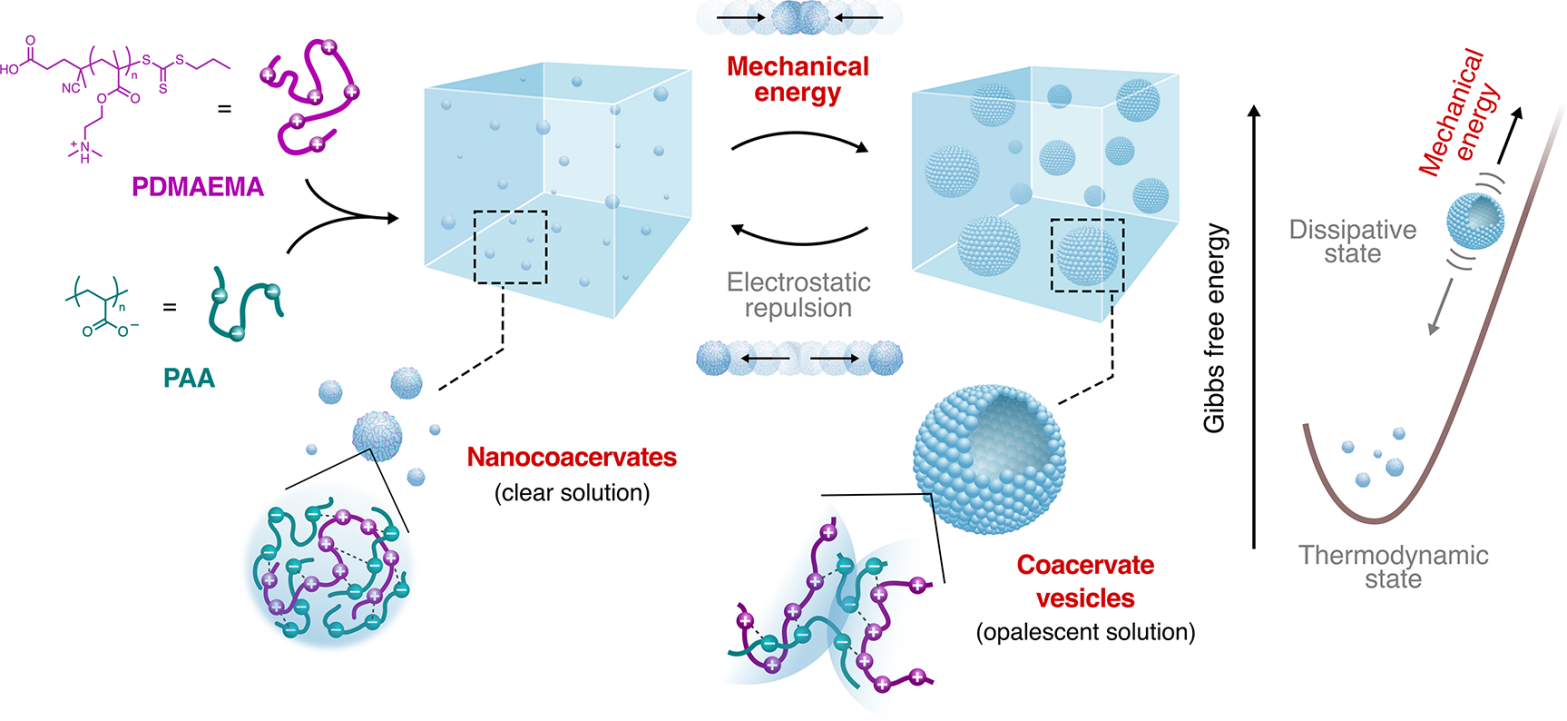
Scheme illustrating the mechanical-force-induced dissipative self-assembly of coacervate vesicles. Initial mixing of the PDMAEMA and PAA polyelectrolyte aqueous solutions (10 mM, pH = 6.5) at a molar ratio of 1:4 produces a stable dispersion of negatively charged nanocoacervates (in a thermodynamic equilibrium state). When an impulsive mechanical force is applied, these nanodroplets reorganize into micrometrical coacervate vesicles (in a dissipative state) held together by inter-nanodroplet polyanion copenetration and electrostatic interactions. Over time, in the absence of mechanical energy input, electrostatic repulsion between nanocoacervate cores gradually drives vesicle disassembly, returning the system to its equilibrium state (*vide infra*).

We then show that these dissipative coacervate vesicles, unprecedented in their mechanical formation and nanostructure, can be chemically programmed to exhibit essential cell-like behaviors, including selective molecular uptake, enzymatic metabolism, and communication based on diffusible chemical signals. Importantly, by exploiting their dissipative nature, we showcased that mechanical energy can also drive compartment evolution, ultimately leading to protocells with spatial enzyme organization and enhanced metabolic functionality.

Overall, our work establishes mechanical energy as a fundamental driver of dissipative self-assembly, opening new avenues for engineering dynamic, cell-like synthetic systems with tunable functionality and metabolism. By harnessing impulsive forces to direct protocell formation and evolution, we advance the frontier of nonequilibrium synthetic biology, offering a powerful strategy to design next-generation life-like materials.

## Results and Discussion

Coacervate vesicles were self-assembled by providing an impulsive force to a dispersion of nanocoacervates formed by mixing aqueous solutions of poly­(2-(dimethylamino)­ethyl methacrylate) (PDMAEMA, 20 kDa, number of monomers per chain ≈127, 10 mM, pH 6.5) and poly­(acrylic acid) (PAA sodium salt, 2.1 kDa, number of monomers per chain ≈22, 10 mM, pH 6.5) (Figure 1). While at near-equimolar ratios, mixtures of aqueous solutions of PDMAEMA and PAA (10 mM, pH 6.5) typically form nearly neutral coacervate *micro*droplets (phase diagram in Figure 2a, yellow region),^49^ we found that coacervate *nano*droplets with an average diameter of 116 ± 40 nm (by dynamic light scattering—DLS) and a zeta potential of −44.0 ± 5.9 mV formed at a 1:4 PDMAEMA/PAA molar ratio (Figure 2b, Figure S8). These negatively charged nanocoacervates are thermodynamically stable over time, showing no changes in zeta potential or signs of coalescence for a long time (Figure S8).

**Figure 2:**
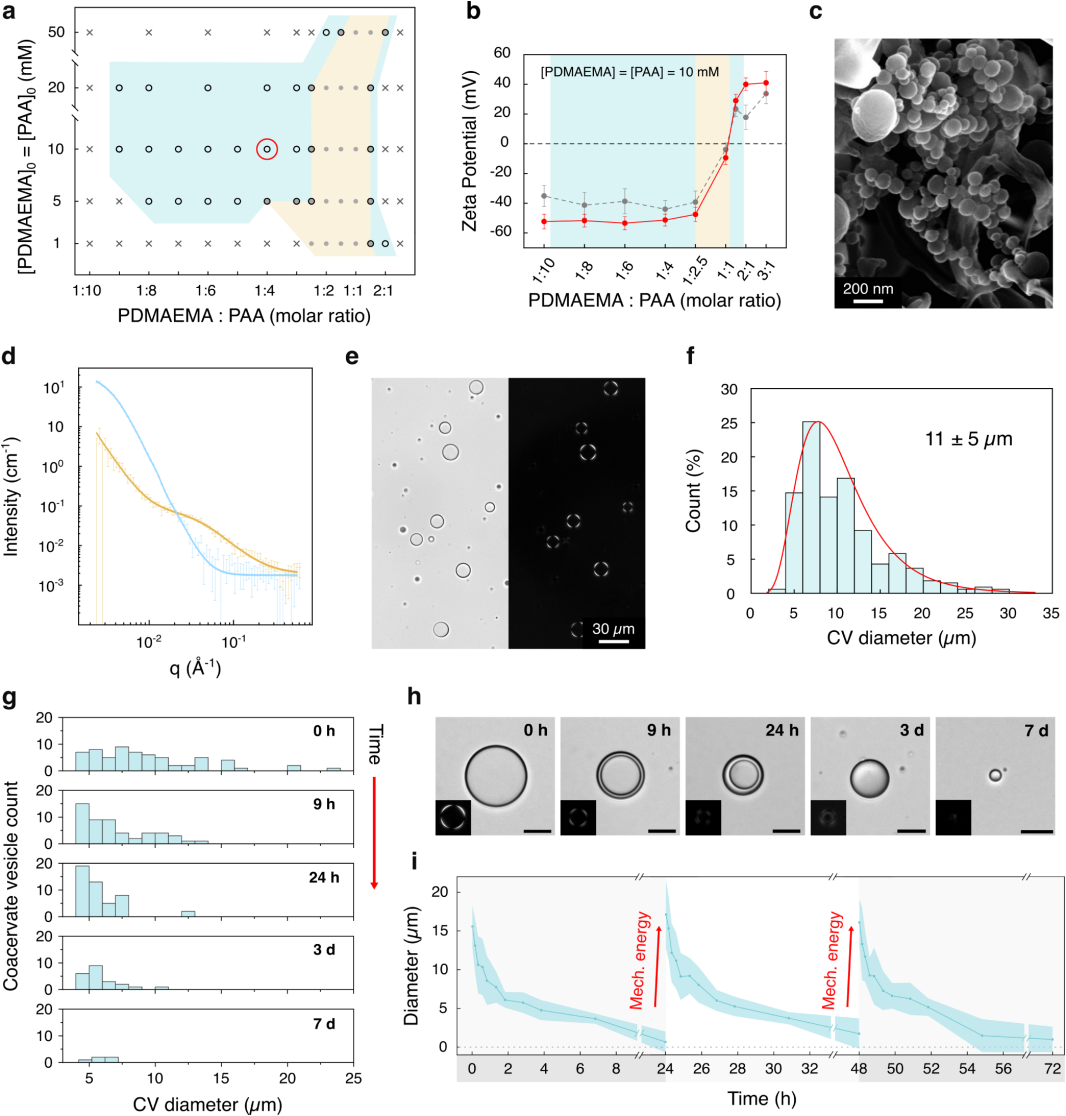
Characterization and time-dependent disassembly of coacervate vesicles. (a) Phase diagram of the PDMAEMA/PAA system (pH = 6.5) at varying molar ratios and polymer concentrations. The yellow region denotes conditions favoring coacervate microdroplet formation; azure region shows conditions enabling coacervate vesicle formation upon 30 s of impulsive force application. The white region shows no coacervation or vesicle formation after impulsive force application. The red circle marks the primary concentration and molar ratio used in this work. (b) Zeta potential measurements comparing nanocoacervates (gray plot) and coacervate vesicles (red plot) assembled from 10 mM PDMAEMA/PAA solutions at different molar ratios. Charge inversion occurs at a 1:1 molar ratio. The yellow area indicates coacervate microdroplet formation, while the azure areas indicate coacervate vesicle formation. Error bars show the standard deviation (*n* = 3). (c) Cryo-SEM image of nanocoacervates formed by mixing PDMAEMA and PAA (10 mM) at a 1:4 molar ratio, showing diameters consistent with DLS measurements. (d) SANS analysis comparing 1:1 (yellow plot, coacervate microdroplets) and 1:4 (azure plot, coacervate nanodroplets) PDMAEMA/PAA (10 mM) molar ratio solutions after 30 s of shaking. (e) Brightfield (left) and cross-polarized (right) microscopy images of coacervate vesicles formed at a 1:4 PDMAEMA/PAA (10 mM) molar ratio. (f) Size distribution analysis of coacervate vesicles formed at a 1:4 PDMAEMA/PAA (10 mM) molar ratio, showing an 11 ± 5 μm average diameter. (g) Time-dependent vesicle disassembly profile. Vesicles initially exhibit a broad size distribution, followed by a reduction to ca. 5 μm after 24 h due to internal lumen depletion. Membrane collapse occurs after 3 days, leading to complete vesicle dissolution after day 7. (h) Brightfield microscopy showing key stages of vesicle disassembly. Images demonstrate progressive membrane thickening until complete collapse on day 3, with only scattered small droplets remaining by day 7, before complete disappearance. The cross-polarized microscopy insets show how the characteristic birefringence of coacervate vesicles is lost during membrane collapsing. Scale bars: 10 μm. (i) Time-dependent changes in the vesicle diameter. After complete dissolution, vesicles reformed upon reapplication of an impulsive force, demonstrating process reversibility. Acceleration of the disassembly process from 7 days to 24 h was achieved using a tube revolver to keep the sample under gentle continuous mixing. Error bands show standard deviation (*n* = 25).

Cryo-scanning electron microscopy (Cryo-SEM) imaging revealed that distinct, noncoalescing spherical nanodroplets ca. 100 nm in diameter formed at a 1:4 PDMAEMA/PAA ratio (Figure 2c), consistent with the DLS and zeta potential results, and contrasting with the coalesced micrometrical droplets observed at a 1:1 PDMAEMA/PAA ratio (Figure S9). The structural differences between these polyion ratios were further confirmed by small-angle neutron scattering (SANS). At a 1:4 PDMAEMA/PAA ratio, the data could be well described by a core–shell oblate or sphere model, indicating nanocoacervates with a solvated PAA-rich shell of ca. 60 nm thick and an internal complex core of a radius of ca. 40 nm (Figure 2d blue plot, Supplementary Section 4, Figure S10). This structural model is consistent with the negative surface charge measured by the zeta potential and with the hydrodynamic diameters obtained from DLS.

These characteristics stand in marked contrast to the 1:1 ratio microdroplets (Figure 2d yellow plot, Figure S10), which exhibit strong clustering upon shaking, with overlapping polymer chains in conformations similar to those in semidilute solutions with a correlation length (or a mesh size) of 2.17 ± 0.04 nm, consistent with Spruijt *et al*.‘s findings.^50^

Importantly, when we applied an impulsive force (1000–4000 Pa) through ca. 30 s of vigorous manual shaking to the 1:4 PDMAEMA/PAA ratio nanodroplet solution (see Supplementary Sections 5 and 8 for detailed protocols on applying the impulsive mechanical force and estimation of the applied force, respectively), we observed the presence of self-assembled organized vesicles with a mean diameter of 11 ± 5 μm and a zeta potential of −51.3 ± 4.0 mV (Figure 2b,e,f). These structures exhibited a distinctive Maltese-cross birefringent pattern (Figure 2e, Figure S12) similar to that observed in other stabilized coacervate-based microcompartmentalized systems such as liquid crystals or polyoxometalate coacervate vesicles,^51,52^ indicating an organized and packed membrane matrix.^53^ While the vesicles gradually disassembled over 7 days, they were reproducibly reformed multiple times from the same solution, demonstrating a reversible mechanical force-mediated dissipative self-assembly process (Figure 2g,h,i, Supplementary Section 6, Figure S13).

Disassembly occurred in distinct stages: first, the diameter gradually decreased over the initial 24 h, and then the internal aqueous lumen collapsed to form a small sphere after approximately 3 days. Finally, after about 7 days, the system completely reverted to its initial thermodynamic equilibrium state by dissolving back into a coacervate nanodroplet solution (Figure 2h, Figure S13). Cryo-SEM analysis provided key structural insights into coacervate vesicles formed at a 1:4 PDMAEMA/PAA molar ratio (10 mM). The images revealed a hollow structure with a distinctive membrane ca. 100 nm thick, characterized by a rough surface resulting from the partial fusion of nanocoacervates (ca. 100 nm in diameter) arranged in a monolayer (Figure 3a, Figure S9). This membrane architecture suggests a formation mechanism where polymer chains interpenetrate to mediate nanocoacervate fusion while allowing each droplet to maintain some of its original structure. The preservation of this molecular organization aligns with our SANS analysis, which demonstrated that the structural arrangement of polymers in the 1:4 PDMAEMA/PAA system (10 mM) underwent no to minimal change even after mechanical perturbation (Figure 2d solid blue curve, Figure S10).

**Figure 3:**
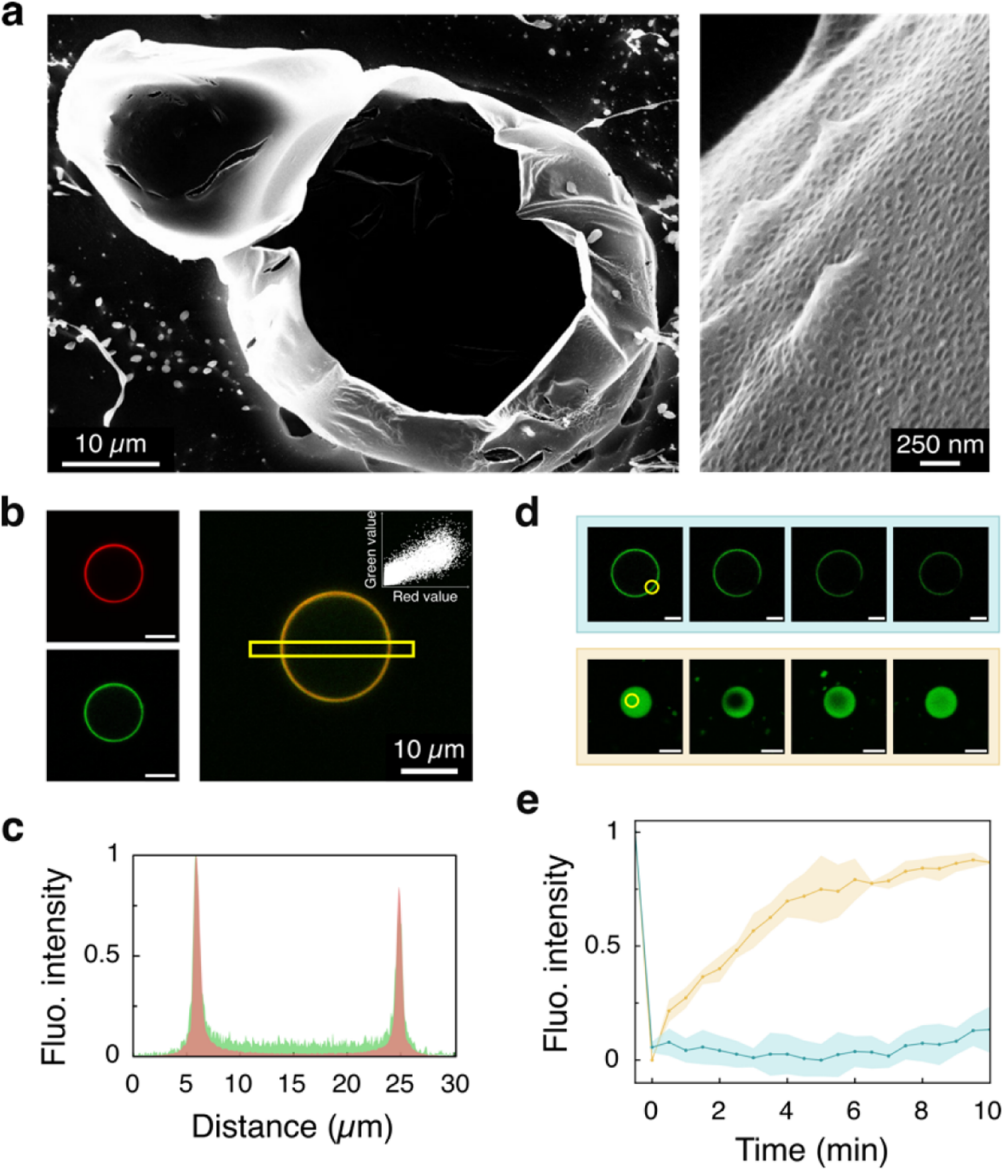
Structural characterization of coacervate vesicles formed from a 1:4 PDMAEMA/PAA (10 mM) molar ratio. (a) Cryo-SEM image of a sectioned coacervate vesicle, revealing its hollow structure. The inset shows a magnified view of the membrane, comprising self-assembled and interpenetrated nanocoacervates ca. 100 nm in diameter. (b) Confocal laser scanning microscopy images of a single coacervate vesicle assembled at a 1:4 ratio of PDMAEMA/PAA (10 mM). The polymer solutions contained 10 mol % FITC-tagged PDMAEMA (green) and 10 mol % RITC-tagged PAA (red), respectively. The merged image (right) shows polymer colocalization, confirmed by the pixel data scatter plot inset (Figure S14). Scale bars = 10 μm. (c) Fluorescence intensity profile (normalized) along the yellow square area in (b) for the red and green channels. (d) FRAP experiment: confocal laser scanning microscopy images of coacervate vesicles (top) and coacervate microdroplets (bottom). Images show pre-excitation, *t* = 0 s, 2 min, and 10 min post-photobleaching. The yellow area indicates the bleached region. Scale bars: 5 μm. (e) Fluorescence recovery curves (normalized) from (d) for coacervate microdroplets (yellow plot) and coacervate vesicles (azure plot) over 10 min post-photobleaching. Vesicles show no recovery due to the low polymer membrane mobility. Error bands show standard deviation (*n* = 3).

Using fluorescently labeled polymers (fluorescein-tagged PDMAEMA, abbr. FITC-PDMAEMA; rhodamine B-tagged PAA, abbr. RITC-PAA, Supplementary Section 2), we examined vesicle structure, composition, and dynamics using confocal laser scanning microscopy. Figure 3b,c confirms that the vesicles are hollow and that there is a strong colocalization of both polymers in the vesicle membrane (Pearson’s coefficient = 0.851, Figure S14), revealing a homogeneous composition of the membrane at the micrometrical level. Fluorescence recovery after photobleaching (FRAP) analysis (Figure 3d,e, Supporting Information, Figure 3d,e, Supplementary Section 7) demonstrated a stark contrast in the polymer chain mobility between standard PDMAEMA/PAA coacervate microdroplets and coacervate vesicles: while coacervates showed rapid fluorescence recovery (*t*_1/2_ ≈ 2 min), vesicle membranes displayed no recovery even after 10 min. This immobility of the polymer chains in the vesicles indicates a tightly packed membrane structure, which corroborated well with Cryo-SEM imaging, SANS analysis, and cross-polarized microscopy birefringence observations.

This remarkable dissipative process of coacervate vesicle formation through impulsive force application was not limited to the 1:4 PDMAEMA/PAA ratio but also occurred across polymer mixtures with varying ratios of polyanion to polycation. The azure area in Figure 2a phase diagram shows all the concentrations that produced coacervate vesicles upon impulsive force application (Figure S15). Notably, vesicle formation occurred at concentrations and ratios just outside the yellow coacervation phase. At 10 mM concentration across the PDMAEMA/PAA ratios of the azure area of the phase diagram, DLS and zeta potential measurements confirmed the presence of nanocoacervates in the starting solution, with sizes ranging between 100 and 200 nm (Figure S16), and zeta potentials ranging from −51.3 ± 4.0 mV to +29.0 ± 4.4 mV as the solution composition shifted from excess PAA to excess PDMAEMA, respectively (Figure 2b, gray plot). The coacervate vesicles formed after impulsive force application retained zeta potential characteristics similar to those of the corresponding nanodroplet building blocks (Figure 2b, red plot).

Overall, these findings led us to propose that the system is governed by a dynamic interplay between mechanical energy and thermodynamics. Impulsive mechanical forces (1000–4000 Pa, Supplementary Section 8) overcome the electrical double layer and osmotic pressure (1500–2200 Pa, Supplementary Section 8) that stabilize the nanocoacervates, bringing them into contact and mediating polymer chain interpenetration and entanglement, resulting in vesicle assembly (Figure 1). Importantly, an impulsive force is needed, and shear forces alone (e.g., stirring, vortexing, or sonication) did not produce a comparable outcome (Figure S11). This process requires excess polyanionic (PAA) or polycationic (PDMAEMA) chains on the nanodroplet surface, explaining why mechanical force-driven self-assembly is observed outside coacervation molar ratios (azure area in the phase diagram of Figure 2a). Interestingly, under highly dilute conditions (1 mM), vesicle formation was not observed with excess polyanion, whereas minimal vesicle formation occurred with excess polycation. This was attributed to the increased spatial separation between nanocoacervates, which reduced collision efficiency and required a too high mechanical energy input for dissipative self-assembly. However, the system’s ultimate fate is determined by thermodynamics: the interpenetration of the dangling chains upon fusion of the nanodroplets would lead to higher monomer concentration and charge density and in turn heightened osmotic pressure in the coacervate membrane; over time, the drive to minimize Gibbs free energy leads to polymer chain disentanglement, causing the vesicles to disassemble and revert to their original state as discrete and stable nanocoacervates.

To demonstrate the generality of the mechanical force-driven dissipative self-assembly of nanocoacervates into vesicles, we investigated both commercially available and bioderived binary polyelectrolyte systems: poly­(diallyldimethylammonium)/poly­(acrylic acid) (PDDA/PAA, Figure 4a) and poly-l-lysine/double-stranded DNA (PLys/dsDNA, Figure 4b). Both systems are known to form coacervate microdroplets at specific molar ratios (Figure 4, yellow regions).^54,55^ Remarkably, upon application of mechanical energy to these systems outside their coacervation phase molar ratios, we successfully generated coacervate vesicles (azure area). Microscopy images further confirmed the formation of birefringent coacervate vesicles in both systems, highlighting the generality of this dissipative self-assembly mechanism and its applicability across various polyelectrolyte systems. Notably, for the PDDA/PAA system, vesicle formation was not observed with an excess of polycation, likely due to the significant molecular weight disparity between PDDA (ca. 100 kDa) and PAA (2.1 kDa), which hindered the interpenetration of the longer PDDA chains.

**Figure 4:**
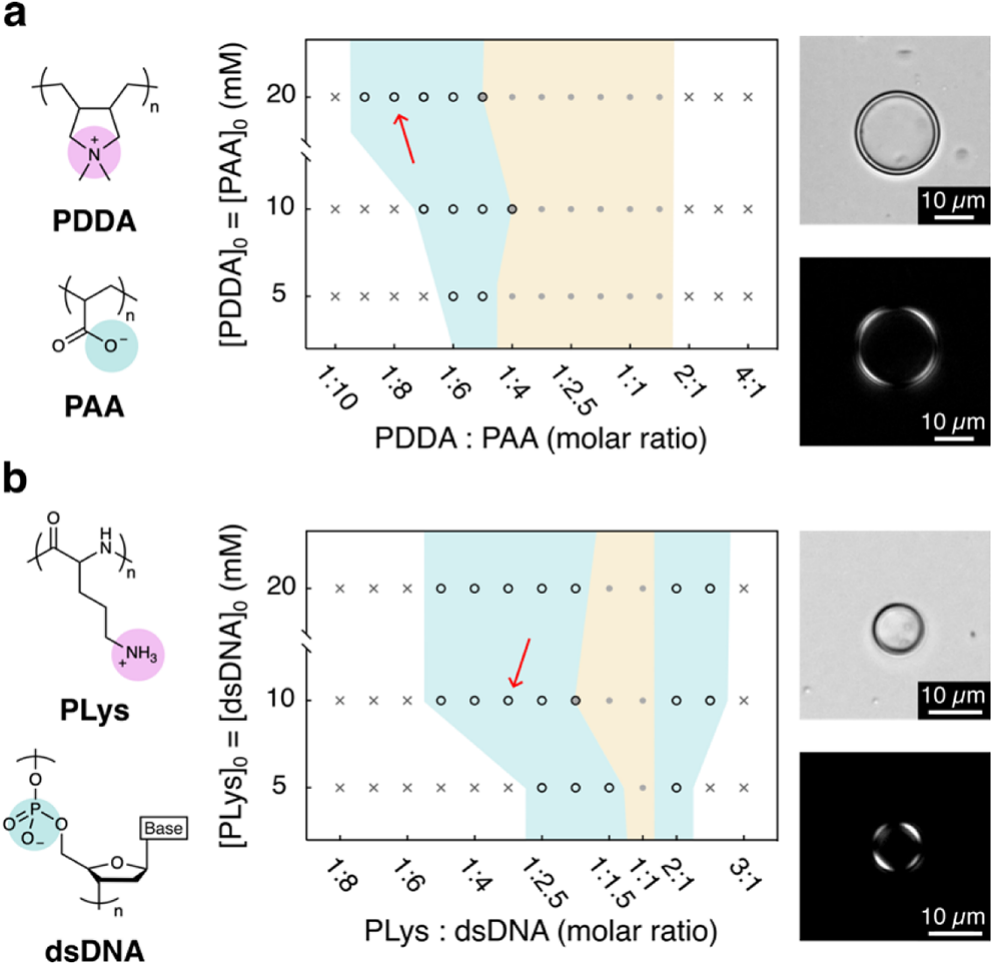
Formation of coacervate vesicles with other polyelectrolyte systems. (a) Molecular structures and phase diagram for the PDDA/PAA system: in the phase diagram, white areas indicate clear solution and absence of structure even after impulsive force application, the yellow area indicates coacervate microdroplets, and azure area indicate vesicles formed after 30 s of manual shaking. The red arrow indicates the conditions (1:8 molar ratio, 20 mM) used to acquire the bright-field (top) and cross-polarized (bottom) microscopy images on the right, showing a typical birefringent single vesicle. The pH of all solutions was adjusted to 6.5. (b) Molecular structures and phase diagram for the PLys/dsDNA system. Color coding for the three different phases is the same as that in (a). The red arrow indicates the conditions (1:3 molar ratio, 10 mM) used to acquire the brightfield (top) and cross-polarized (bottom) microscopy images on the right, showing a typical birefringent single vesicle. The pH of all solutions was adjusted to 6.5.

Next, we investigated whether the coacervate vesicles could selectively sequester and concentrate guest molecules from the external environment.^56^ For this, we assembled coacervate vesicles in the presence of various fluorescent organic dyes and employed confocal laser scanning microscopy to analyze their interaction (Figure 5a, Supplementary Section 9 and Table S4). These experiments revealed that their distribution in coacervate vesicles was influenced by molecular charge (at pH 6.5) and hydrophobicity (Supplementary Section 10). Membrane uptake was primarily governed by hydrophobicity, with dyes exhibiting higher log *D* values (e.g., Nile Blue) showing greater sequestration than those with lower log *D* values (e.g., Eosin Y). In contrast, lumen localization was predominantly influenced by molecular charge, favoring positively charged, weakly hydrophobic molecules (e.g., [Ru­(bpy)_3_]^2+^). Notably, highly negative and hydrophilic dyes (e.g., Calcein) showed negligible vesicular uptake. The ability of these coacervate vesicles to selectively sequester and integrate in specific regions diverse chemical species, including common photocatalysts such as [Ru­(bpy)_3_]^2+^ and Eosin Y, as well as potential drug molecules, highlights their potential as a novel protocell scaffold.

**Figure 5:**
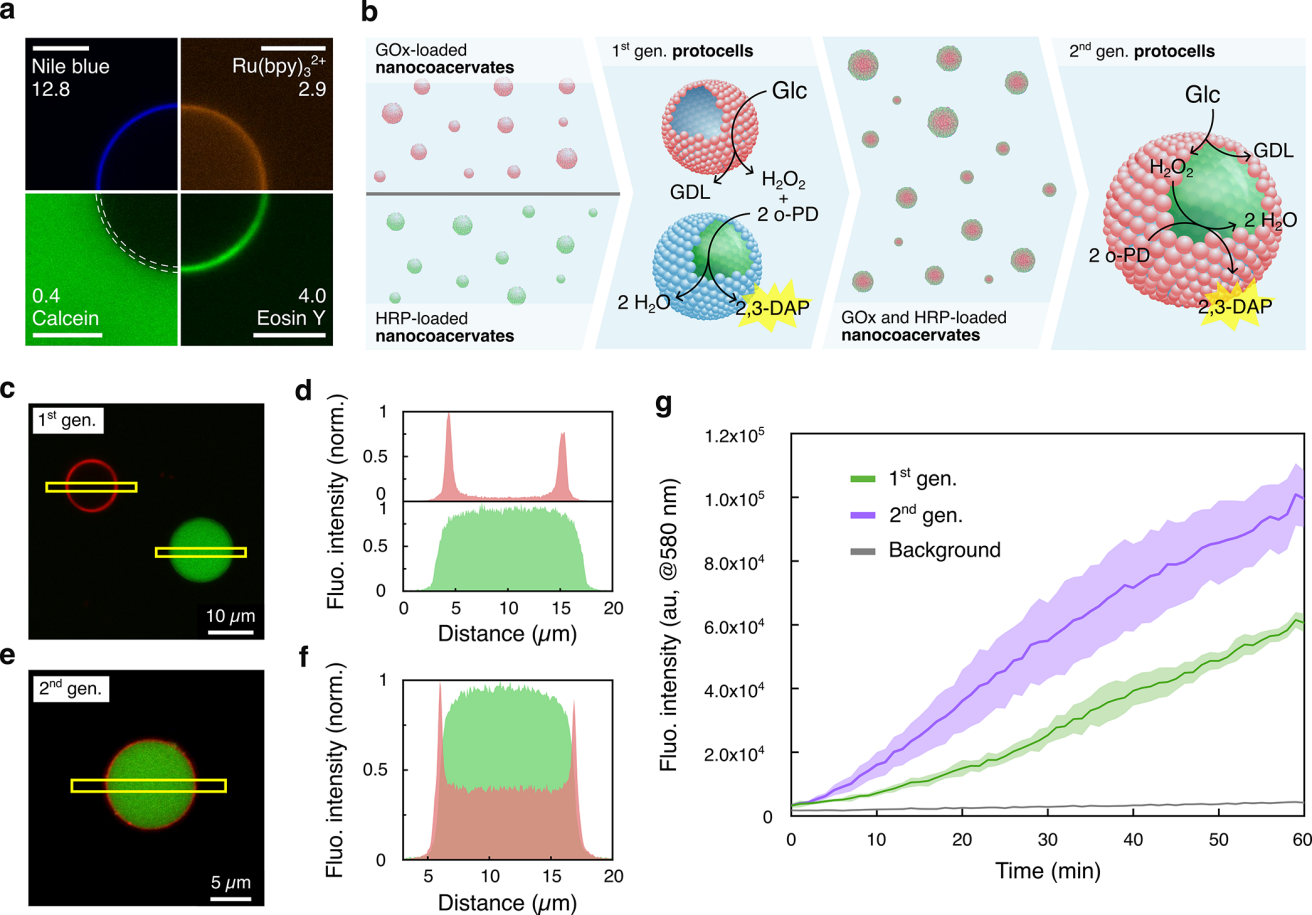
Mechanical energy-mediated evolution of a protocell consortium. (a) Confocal laser scanning microscopy images showing fluorescent molecule uptake by coacervate vesicles (1:4 PDMAEMA/PAA, 10 mM). Membrane equilibrium distribution coefficients are shown next to each dye name. The dashed white lines in the Calcein image indicate the vesicle membrane. Scale bars = 10 μm. (b) Coacervate vesicle preparation and evolution schematic. Initial stage: populations of GOx- (red shapes) or HRP-containing (green shapes) PDMAEMA/PAA nanocoacervates (10 mM, 1:4 ratio) in separate environments. Impulsive force creates two vesicle populations containing either GOx (red, mostly present in membrane) or HRP (green, mostly present in lumen), which are mixed in a 1:1 ratio to form the initial binary protocell consortium (1st generation). After vesicle disassembly, the enzymes randomly distribute in both nanocoacervate populations. Application of impulsive force creates a 2nd generation of vesicles containing both enzymes. The reaction scheme shows the H_2_O_2_ role as a signaling molecule between enzymes. (c) Confocal laser scanning microscopy image of 1st generation coacervate vesicles showing enzyme distribution: RITC-tagged GOx (red) localizes in the membrane, while FITC-tagged HRP (green) concentrates in the aqueous lumen. (d) Fluorescence line profile plots along the yellow areas in (c) demonstrate selective enzyme encapsulation. (e) Confocal laser scanning microscopy image showing enzyme distribution in a single 2nd generation coacervate vesicle. (f) Fluorescence line profile plots along the yellow area in (e). (g) Time-dependent fluorescence intensity (λ_em_ = 580 nm) showing 2,3-DAP production in 1st generation (green plot, initial velocity = 6 ± 1 I_fluo_ s^–1^) and 2nd generation (purple plot, initial velocity = 22 ± 8 I_fluo_ s^–1^) coacervate vesicles against a hydrogel background (gray plot). Error bands show standard deviation (*n* = 3).

Subsequently, we leveraged both the impulsive force-mediated dissipative self-assembly mechanism and their selective sequestration capabilities to endow coacervate vesicles with a rudimentary internalized enzymatic metabolism and chemical communication capabilities based on diffusible chemical signals. We then investigated whether mechanical energy could drive not only the dissipative self-assembly of protocells but also their evolution toward more complex entities with an advanced spatial enzyme organization and enhanced metabolic functions. For this, we started by generating nanocoacervates containing either RITC-tagged glucose oxidase (GOx) or FITC-tagged horseradish peroxidase (HRP) (step I, Figure 5b, Supplementary Sections 11 and 12; GOx and HRP loading was verified using DLS, see Supplementary Figure S20), and used them to self-assemble two different populations of coacervate vesicle-based protocells endowed with a rudimentary enzymatic metabolism.

Interestingly, confocal laser scanning microscopy revealed that these enzymes segregated into different regions: FITC-tagged HRP concentrated in the aqueous lumen of the vesicle, whereas RITC-tagged GOx accumulated in the membrane (Figure 5c,d, Figures S19 and S20). This distribution pattern aligned well with the properties of the two enzymes and the results obtained with the fluorescent organic dyes. GOx, being larger (160 kDa), negatively charged at pH 6.5, and more hydrophobic, behaved similarly to Eosin Y by accumulating in the membrane. Conversely, HRP (44 kDa), being smaller, positively charged, and slightly hydrophilic, concentrated in the lumen like [Ru­(bpy)_3_]^2+^ (Table S5, Figures S20 and S21, Supplementary Section 10).

The two populations of GOx- or HRP-containing protocells were then mixed together in a Petri dish. Confocal microscopy confirmed the presence of these separate protocell populations (Figure 5b, 1st gen.). When left undisturbed, these protocells gradually disassembled over 7 days, reverting to the thermodynamic equilibrium state characterized by enzyme-containing nanocoacervates (Figure 5b, step III). Upon reapplication of impulsive force, a second generation of protocells formed, containing both enzymes within a single microcompartmentalized system (Figure 5b, 2nd gen.). Importantly, the spatial segregation of the two enzymes was maintained, with GOx remaining in the membrane and HRP in the internal lumen (Figure 5e,f, Figure S22). This dynamic cycle of dissipation and reassembly under nonequilibrium conditions demonstrates how mechanical energy can drive protocell evolution, progressing from the assembly of simple and distinct protocell populations to a unified, hierarchically organized system with spatially partitioned enzymes.

To study the chemical communication between the binary population of GOx- or HRP-containing protocells (1st generation) and monitor their evolution into metabolically enhanced second-generation protocells, we embedded both generations in an agarose hydrogel matrix within a cuvette for kinetic fluorescence assays. The hydrogel slowed substrate diffusion, enabling a clear resolution of reactivity differences between protocell generations (Figure S23).

Chemical communication between the binary population of GOx- or HRP-containing protocells was established by introducing glucose and *o*-phenylenediamine (*o*-PD) into the cuvette with the first-generation protocells. The coacervate vesicles actively sequestered the substrates. The GOx-containing protocells oxidized glucose to glucono-δ-lactone (GDL), producing H_2_O_2_ as a diffusible chemical signal. This H_2_O_2_ triggered distal HRP-containing protocells to convert environmental *o*-PD into fluorescent 2,3-diaminophenazine (2,3-DAP) (Figure 5b).

Strikingly, under the same experimental conditions, the second-generation protocell population exhibited a more than three times faster initial 2,3-DAP production rate (Figure 5g). This enhancement arose from their advanced metabolic architecture featuring GOx localized in the membrane and HRP concentrated in the lumen of the same protocell. This spatial organization optimized H_2_O_2_ transfer efficiency between the two enzymes, as further confirmed by the time-dependent pH decrease associated with gluconolactone (GDL) production (Figure 5b), which subsequently hydrolyzes to gluconic acid. Since acid production is associated only with the first half of the enzyme cascade and does not involve diffusion of a signaling molecule, both systems exhibited comparable rates of acid production (Figure S23e). This confirms that the enhanced overall reaction rate in the second-generation system arises specifically from improved H_2_O_2_ transfer rather than differences in GOx activity.

## Conclusions

To conclude, this research demonstrates how mechanical energy can drive the construction of transient cell-like microcompartments through dissipative self-assembly, a previously overlooked mechanism with profound implications for both biological and synthetic systems. The application of mechanical energy to nanocoacervates (116 ± 40 nm) that formed near the bulk coacervation phase boundary triggers their assembly into hollow vesicles 11 ± 5 μm in diameter. These vesicles form through force-mediated interaction of excess polyions on the nanodroplet surfaces and, when left unperturbed, gradually decay back to nanodroplets with a half-life of ca. 2 days. The vesicles retain hallmark properties of complex coacervates, including their ability to concentrate specific molecules and catalysts from their environment, while offering enhanced functionality such as precise molecular segregation between the membrane and lumen.

The synergistic combination of dissipative self-assembly and selective sequestration can then be exploited to establish dynamic behavior within a binary protocell population. We showed that mechanical energy could drive the reorganization of two distinct specialized protocells—one containing the GOx enzyme and the other containing HRP—into a single, evolved higher-order population. This evolution yielded advanced compartmentalization and enhanced coordinated synthetic capabilities that exploited both enzymes within a single protocellular entity. Overall, this mechanical-energy-driven transformation from separated specialized protocells to integrated higher-order entities demonstrates how dissipative self-assembly can also lead to compartment dynamics and more complex protocellular functions.

Finally, we showed that this assembly mechanism worked across different polyelectrolyte systems including DNA and polycationic peptide combinations. Our findings suggest that similar dissipative vesicular structures may exist in nature and play a role in ecosystems and biology. Such dissipative microstructures could have also formed on the early Earth when natural polyelectrolytes^57–60^ encountered mechanical forces from phenomena such as cascades, waves, or underwater eruptions, potentially contributing to the emergence of primitive cells.

From a broader perspective, this study reveals mechanical forces as a critical, previously overlooked driver in forming complex far-from-equilibrium microcompartments with cell-like properties. The discovery that mechanical energy can create dissipative, functional coacervate vesicles represents a paradigm shift beyond current models that utilize chemical fuel or light as the energy source. The implications span multiple fields: for example, in biotechnology, this mechanism could revolutionize drug delivery by enabling the direct self-assembly of DNA- or RNA-based drugs into vesicular carriers through simple vial agitation, potentially eliminating the need for additional carriers. Moreover, the versatility of the system across different polyelectrolyte combinations, coupled with its ability to selectively sequester and organize catalysts and reactivity, provides unprecedented insights into the origin of primordial microcompartmentalized systems and complex molecules. Our findings bridge a crucial gap in dissipative self-assembly research while simultaneously advancing frontiers in protocell engineering, supramolecular chemistry, soft matter chemistry and physics, microbioreactor technologies, drug delivery, and origin-of-life research.

## Supplementary Material



## Data Availability

All data needed to evaluate the conclusions in this study are present in the main text or the Supporting Information. Data obtained at ISIS are available at: 10.5286/ISIS.E.RB2420467.

## Notes

Materials and instruments; details of methods; synthesis and characterization of molecules and polymers; additional experimental details and discussions (DOCX)
