# Supplementary material for: Mechanical Energy Drives Dissipative Self-Assembly of Nanocoacervates into Vesicles with Cell-like Properties
Source: J Am Chem Soc. 2025 Dec 23;148(2):2273–82. doi: 10.1021/jacs.5c14198 (PMC12833805; doi:10.1021/jacs.5c14198)
Supplement: Supplementary file 1 [file ja5c14198_si_001.docx]

Supplementary Information

**Mechanical Energy Drives Dissipative Self-Assembly of Nanocoacervates
into Vesicles with Cell-Like Properties**

**Authors:** Francesco Vicentini^1,2^, Aina Rebasa-Vallverdu^1,2^, Martina Conti^3^, Simone Dal Zilio^3^, Aharon Steffè^1^, Wuge H. Briscoe^4^ *, Pierangelo Gobbo^1,2^ *

**Affiliations:**

1. Department of Chemical and Pharmaceutical Sciences, University of Trieste; Via L. Giorgieri 1, 34127, Trieste (Italy).

2. National Interuniversity Consortium of Materials Science and Technology Unit of Trieste; Via G. Giusti 9, 50121, Firenze (Italy).

3. CNR-IOM - Istituto Officina dei Materiali, Consiglio Nazionale delle Ricerche; Area Science Park, Basovizza, Strada Statale 14, Km 163,5, 34149 Trieste (Italy).

4. School of Chemistry, University of Bristol; Cantock’s Close, Bristol BS8 1TS (United Kingdom).

* Email: [pierangelo.gobbo@units.it](mailto:pierangelo.gobbo@units.it), wuge.briscoe@bristol.ac.uk

Table of Contents

[1. Supplementary Methods 3](#_Toc216778905)

[2. Synthesis and characterization of molecules and polymers 6](#_Toc216778906)

[3. Generation of phase diagrams 17](#_Toc216778907)

[4. Small-angle neutron scattering (SANS) experiments 20](#_Toc216778908)

[5. Preparation of coacervate vesicles 23](#_Toc216778909)

[6. Reversible generation of coacervate vesicles 25](#_Toc216778910)

[7. Fluorescence recovery after photobleaching (FRAP) experiments 28](#_Toc216778911)

[8. Nanocoacervates fusion based on impulsive pressure 31](#_Toc216778912)

[9. Retention and uptake studies 33](#_Toc216778913)

[10. Supplementary discussion of molecular uptake results 36](#_Toc216778914)

[11. Enzyme labelling 37](#_Toc216778915)

[12. In-vitro evolution of a binary population of coacervate vesicles 37](#_Toc216778916)

[13. Hydrogel preparation and enzyme cascade reaction kinetic studies 44](#_Toc216778917)

1. Supplementary Methods

All solvents were purchased in analytical grade from Sigma-Aldrich and used without further purification. Milli-Q water was obtained from a Millipore Milli-Q Plus 185 apparatus and presented a resistivity of 18.2 MΩcm. All reagents were used as received unless otherwise stated. Poly(acrylic acid sodium salt) (PAA; 2.1 kDa), poly(diallyldimethylammonium chloride) solution (< 100 kDa; PDDA), deoxyribonucleic acid from salmon sperm (dsDNA; low molecular weight), poli-L-lysine hydrobromide (PLys; 15 – 30 kDa), glucose oxidase (GOx) (252100 U mg^-1^), horseradish peroxidase (HRP) (172 U mg^-1^), fluorescein isothiocyanate (FITC), rhodamine B isothiocyanate (RITC), *o*-phenyldiamine (*o*-PD), α-D-glucose (Glc), propanethiol, sodium hydroxide, carbon disulphide, tosyl chloride, 4,4-azobis(4-cyanovaleric acid) (ACVA), 2,2’-azobis(2-metilpropionitrile) (AIBN), *N*,*N-*(dimethylamino)ethyl methacrylate (DMAEMA), acrylic acid (AA), fluorescein O-methacrylate, multipurpose agarose, and PBS Tablets were purchased from Sigma-Aldrich. Methacryloxyethyl thiocarbamoyl rhodamine B was purchased from Polysciences.

^1^H and ^13^C NMR spectra were recorded on a Jeol GX-400 MHz spectrometer. ^1^H NMR spectra are reported as δ in units of parts per million (ppm) relative to chloroform (δ 7.26, s) and water (δ 4.79, s). The number of protons (*n*) for a given resonance is indicated as *n*H and was based on spectral integration values. ^13^C NMR are reported as δ in units of parts per million (ppm) relative to CDCl_3_ (δ 77.16, t).

Dynamic light scattering and zeta potential measurements were performed on a Zetasizer Nano series instrument (Malvern Instruments, UK). Samples were dissolved in an appropriate aqueous solvent for the different specific experiments, and the solutions were filtered using Acrodisc 0.2 μm nylon filters and disposable Malvern Panalytical folded capillary zeta cells were used to conduct the analysis.

UV-vis spectroscopy was conducted using a Cary 5000 UV-Vis-NIR spectrophotometer, in the wavelength range of 175–900 nm. The instrument was equipped with tungsten-halogen lamps for the visible range and deuterium arc lamps for the UV range. Samples were prepared by dissolving the specified amount of analyte in Milli-Q water.

Attenuated total reflectance Fourier transform infrared (ATR-FT-IR) spectra were recorded using a Shimadzu IR Affinity-1S Fourier Transform Infrared spectrophotometer. Polymer samples were measured directly as solids, and the blank was automatically subtracted from each spectrum. The data were processed and analyzed using LabSolutions IR software.

A Seven Compact digital pH-meter by Mettler Toledo was used for pH determinations. The pH electrode was an InLab Semi-Micro glass electrode. The pH meter was calibrated before each set of measurements using standard buffer solutions at pH 4.01, 7.00, and 10.01, ensuring accurate readings across the relevant pH range.

Fluorescence spectroscopy measurements were performed on an Edinburgh instruments FS5 spectrofluorometer using a 150 W CW Ozone-free xenon arc lamp as source and a Photomultiplier R928P (spectral coverage 200 nm – 900 nm, cooled and stabilized) as the detector. 1 mL Hellma quartz cuvettes with a 10 mm path length were used for these experiments.

The degree of polymerization (DP) of the polymers synthesized in this work was determined from the ^1^H NMR spectra of the polymerization solution taken before and after the polymerization reaction. Dimethylformamide (DMF) was used as an internal standard. The DP was calculated from the ratio of the integrals of the monomer at the end of the polymerization reaction to those before the polymerization reaction was initiated (*t_0_*).

The determination of the molecular weight of the synthesized polymers was performed with a Malvern OMNISEC chromatography system configured with a quadruple detector system (GPC). The OMNISEC REVEAL detector system consisted of a differential refractometer (*l* = 640 nm), a UV-vis diode array spectrometer (*l* = 190–900 nm), a light scattering detector with right angle light scattering (RALS) operating at 90° and low angle light scattering (LALS) at 7° (*l* = 640 nm), and a differential viscometer comprising four capillaries, a delay column, and two pressure transducers (DP and IP). The detectors were arranged in the following order: RI – UV – RALS – LALS – viscosimeter. All detectors were calibrated with pullulan and dextran in an aqueous mobile phase. The OMNISEC RESOLVE system included pre-installed mobile phase pumps, an autosampler with a 300 μL injection loop, a degasser, and SEC columns. OMNISEC v11.41 software was used for SEC data acquisition and analysis. A 0.2 μm cellulose acetate filter was placed between the columns and the detectors. The eluent was filtered using a 0.1 μm Omnipore PTFE filter. The delays between the detectors and the band broadening parameters were automatically calculated by the OMNISEC software using pullulan as the narrow standard.

Optical and fluorescence microscopy was performed on a Axio Observer 7 (Zeiss) microscope at 20× and 40× magnification. The microscope was equipped with an Orca Flash 4.0 V3 CMOS camera (Hamamatsu) and a motorized XYZ sample stage. The brightfield images were obtained using a transmitted light LED lamp. The birefringence images were acquired using crossed polarizer filters. Images were analyzed using the last version of ImageJ software.

Confocal laser scanning microscopy images were obtained on a FV3000 (Olympus) confocal laser scanning microscope. The system was equipped with 4 excitation laser lines: 405, 488, 561, and 640 nm (Coherent), a galvanometric scanning head, and 4 spectral detectors, allowing for simultaneous imaging of up to 4 fluorescence channels. Brightfield images were acquired using a scanning laser line and a transmitted light detector. The inverted microscope body (Olympus IX 83) included a motorized automated sample stage for multi-area tiled imaging. The following objectives were used: UPLXAPO 20×/0.8 NA (Olympus), and an oil objective UPLXAPO 60×/1.42 NA (Olympus). Images were taken using excitation wavelengths of 405 nm (o-PD), 488 nm (FITC) and 561 nm (RITC) and emission wavelengths of 550-625 nm, 500-550 nm and 575-650 nm, respectively. Images were analyzed using the last version of ImageJ software.

For the scanning electron cryo-microscopy (Cryo-SEM) analysis the liquid samples were placed in the holders of a custom-made ZEISS shuttle and transferred to a Leo Crossbeam 1540XB FIB-SEM (ZEISS Microscopy, Oberkochen, Germany) using a QUORUM PP3010Z cryo-stage with a special transfer system. The samples were rapidly frozen at -190 °C, followed by sublimation at -90 °C for 20 minutes to remove surface water. Subsequently, the samples were sputtered with platinum at a current of 10 mA for 30 s. To obtain vesicles cross-sections, the frozen samples were fractured and cut open with a scalpel before sublimation inside the cryo-stage. The temperature was maintained at -175 °C throughout the imaging process. Images were captured using Zeiss SmartSEM software and analyzed using the last version of ImageJ software.

Small-angle neutron scattering (SANS) experiments were conducted on the SANS2d instrument at the ISIS Neutron Source (STFC Rutherford Appleton Laboratory) in Oxfordshire, UK. A simultaneous Q-range of 0.005 – 1.0 Å^-1^ was achieved utilizing an incident wavelength range of 1.75 – 16.5 Å. The beam is centered on the 96.5 × 96.5 cm^2^ detector, with a fixed distance of 8 m between the sample and the detector. The beam size was 8 mm × 8 mm. The solutions were contained in Hellma quartz spectrophotometer cuvettes with a path length of 2 mm and were maintained at a temperature of 25 ± 1°C. The scattering from the empty cell and solvent (D_2_O) was subtracted from the raw data. The data were reduced using Mantid and analyzed with the latest version of SasView.

1. Synthesis and characterization of molecules and polymers
   1. **Synthesis of 4-cyano-4-[(propylsulfanylthiocarbonyl)sulfanyl]pentanoic acid (CPP)**

CPP was synthesized following a previously published procedure, which was slightly modified*.*^1^ Propanethiol (0.076 g, 1.0 mmol) was dissolved in a 1:1 mixture of acetone and water (8 mL) with sodium hydroxide (0.10 g, 2.0 mmol). After cooling to 0°C, carbon disulfide (0.092 g, 73 μL, 1.2 mmol) was added dropwise while stirring. After 2 hrs the reaction mixture was cooled to –5°C with an acetone/ice bath, and a tosyl chloride (0.23 g, 1.2 mmol) solution in acetone (2 mL) was added dropwise, and the reaction was stirred for a further 1 hr at room temperature. The reaction mixture was acidified to pH 2 with HCl (1 M) and extracted with ethyl acetate (2 x 20 mL). The solution was then concentrated to *ca.* 10 mL by rotary evaporation and ACVA (0.56 g, 2.0 mmol) was added, and the mixture was stirred under reflux at 80°C overnight. The crude was purified by column chromatography without workup, over silica, using ethyl acetate/hexane (1:3) + 1% (v/v) acetic acid as the eluent. The product was a yellow oil (0.20 g, 0.72 mmol, yield = 72 wt%).

^1^H NMR (400 MHz, CDCl_3_): δ (ppm) = 3.32 (t, J = 7.2 Hz, 2H, SCH_2_), 2.74 – 2.61 (m, 2H, CH_2_COOH), 2.60 – 2.33 (m, 2H, CH_2_CH_2_COOH), 1.88 (s, 3H, CH_3_), 1.74 (h, J = 7.3 Hz, 2H, CH_3_CH_2_), 1.02 (t, J = 7.3 Hz, 3H, CH_3_CH_2_).

^13^C NMR (101 MHz, CDCl_3_): δ (ppm) = 217.0, 176.7, 119.0, 46.4, 39.0, 33.7, 29.6, 25.0, 21.4, 13.6.


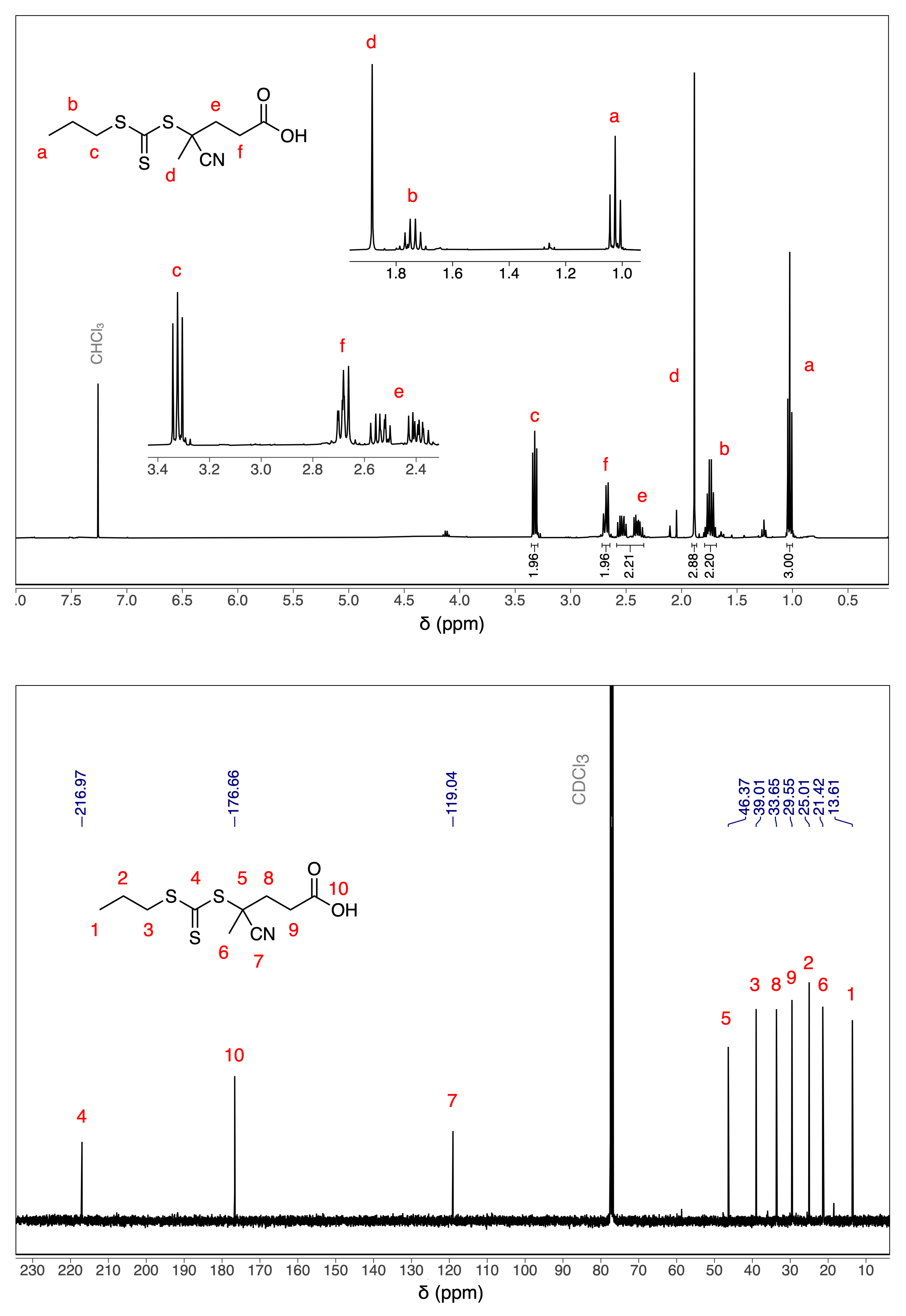


**Fig. S1.** Top: ^1^H NMR spectrum of CPP acquired in CDCl_3_ and referenced against residual CHCl_3_. Bottom: proton decoupled ^13^C NMR spectrum of CPP acquired in CDCl_3_ and referenced against CDCl_3_.

- 1. **Synthesis of poly(2-(dimethylamino)ethyl methacrylate) (PDMAEMA)**

AIBN was freshly recrystallized from methanol, DMAEMA monomer was purified from the inhibitor through a basic alumina column. DMAEMA (0.486 g, 3.09 mmol), AIBN (1.31 mg, 8.00 μmol) and CPP (11.1 mg, 40.0 μmol) were dissolved in 1 mL of 1,4-dioxane in a Schlenk tube equipped with a stirrer bar. DMF (0.1 mL) was added as an internal standard to calculate the degree of polymerization. After sealing the tube, the solution was degassed with four freeze-pump-thaw cycles and refilled with argon for 30 min. Polymerization was carried out at 65°C for 14 hrs. The polymer was isolated by precipitation from cold hexane (50 mL), and subsequently it was lyophilized to obtain a light-yellow solid in 70 wt% yield. ^1^H NMR (400 MHz, CDCl_3_): DP = 80%. GPC: 0.1 M NaNO_3_ at pH 2.6 (CH_3_CO_2_H); *M_n_* (GPC) = 19,677 gmol^−1^, *Đ* (GPC) = 1.128, calculated *dn*/*dC* = 0.192.

For the GPC analysis, the stationary phase consisted of a Tosoh TSKgel G5000 cationic aqueous solvent column (300 mm length, 7.8 mm ID) and a Tosoh TSKgel G3000 cationic aqueous solvent column (300 mm length, 7.8 mm ID) connected in series. The two chromatography columns were preceded by TSKgel PWXL guard column (40 mm length, 6 mm ID). Isocratic elution was performed with a flow rate of 0.5 mL/min, and 50 μL of sample at a concentration of 3 mg/mL was injected.


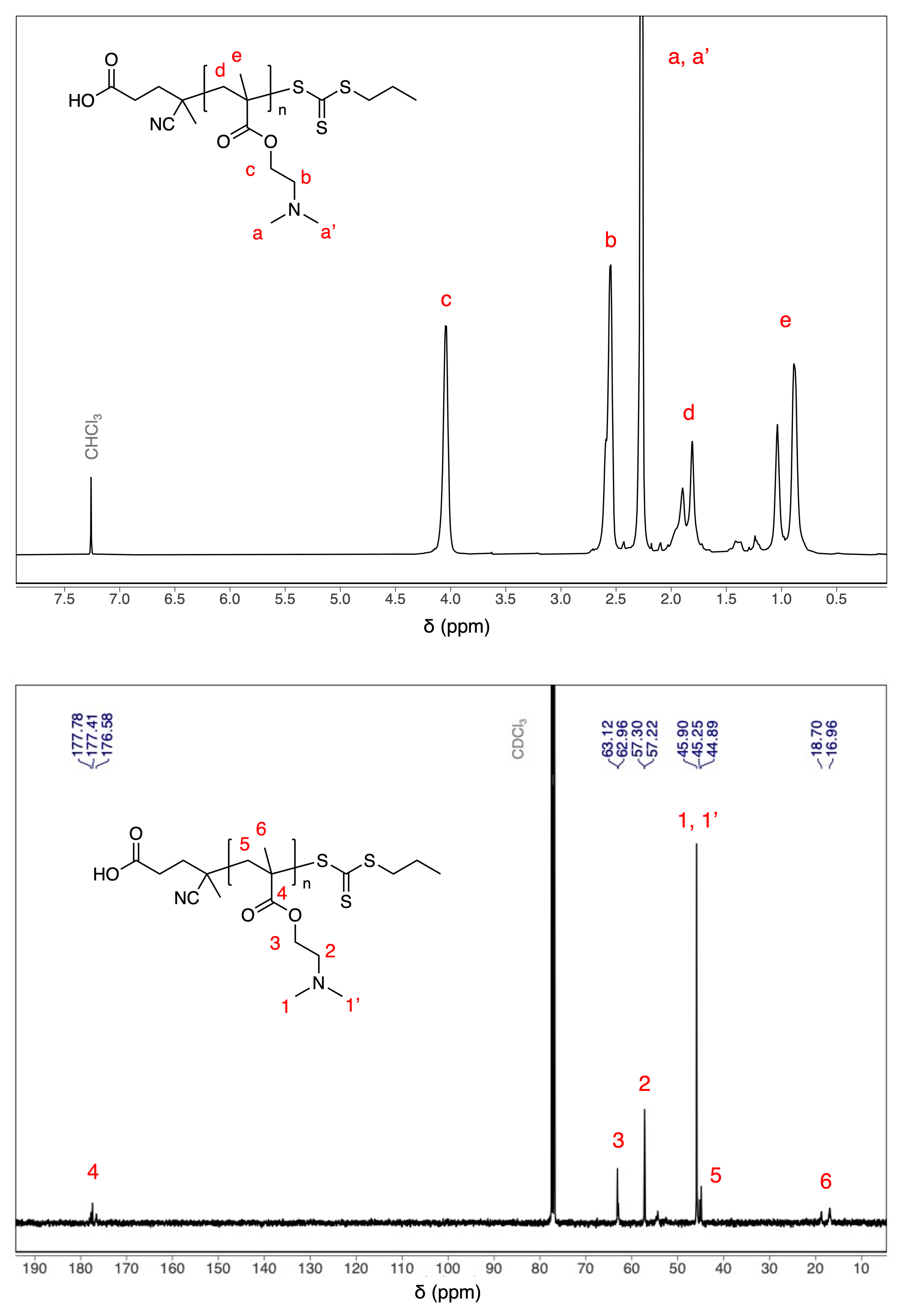


**Fig. S2.** Top: ^1^H NMR spectrum of PDMAEMA acquired in CDCl_3_ and referenced against residual CHCl_3_. Bottom: proton decoupled ^13^C NMR spectrum of PDMAEMA acquired in CDCl_3_ and referenced against residual CDCl_3_.


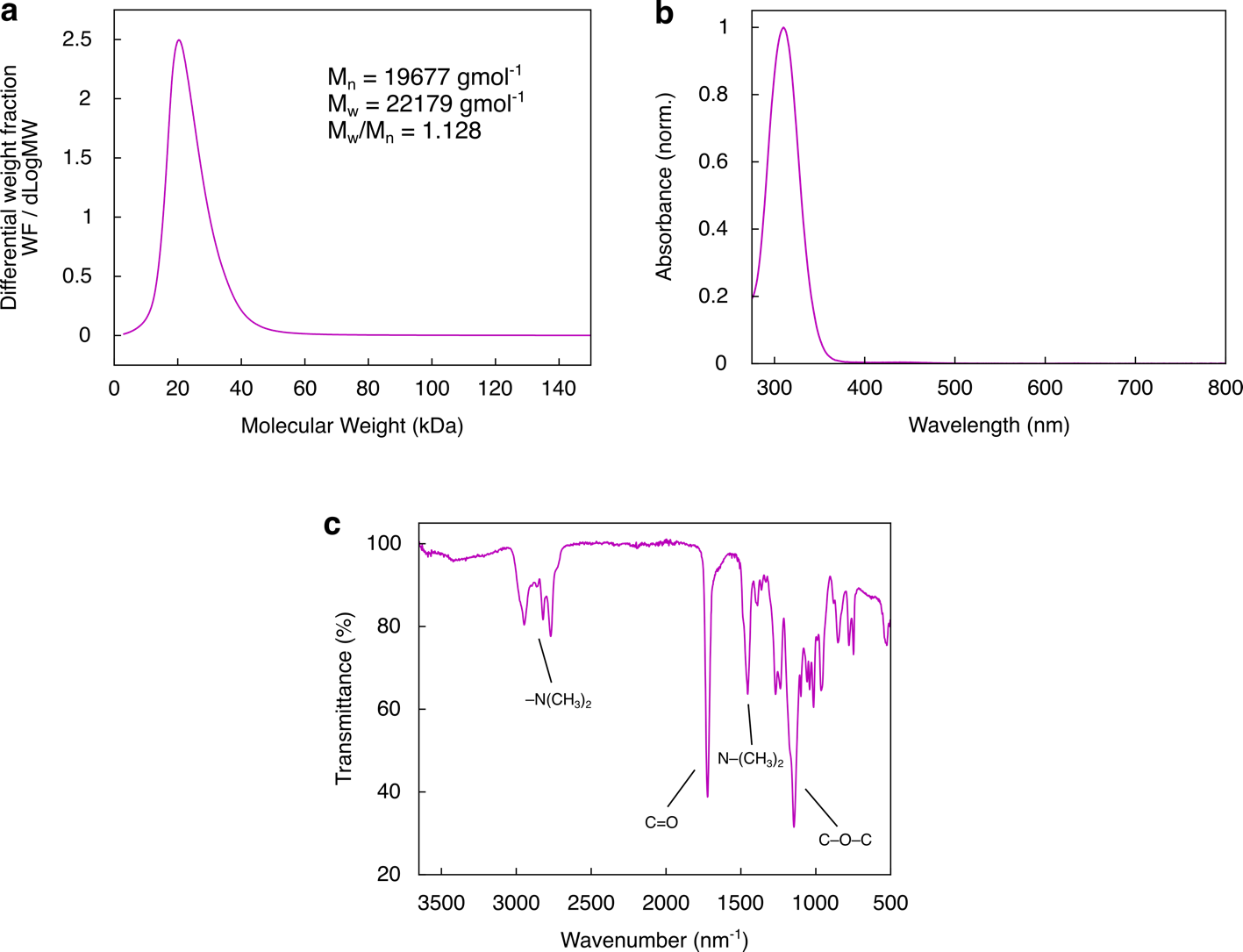


**Fig. S3.** Characterization of PDMAEMA. **a,** GPC molar mass distribution. **b,** Normalized UV-vis spectrum acquired in Milli-Q water at a concentration of 1 mg mL^-1^. **c,** ATR-FT-IR spectra of PDMAEMA.

- 1. **Synthesis of poly(2-(dimethylamino)ethyl methacrylate)-co-fluorescein-O-methacrylate (FITC-PDMAEMA)**

AIBN was freshly recrystallized from methanol, DMAEMA monomer was purified from the inhibitor through a basic alumina column. DMAEMA (0.499 g, 3.17 mmol), fluorescein *O*-methacrylate (0.51 mg, 1.30 μmol), AIBN (1.31 mg, 8.00 μmol) and CPP (11.1 mg, 40.0 μmol) were dissolved in 1 mL of 1,4-dioxane in a Schlenk tube equipped with a stirrer bar. DMF (0.1 mL) was added as an internal standard to calculate the degree of polymerization. The tube was sealed, the solution was degassed with four freeze-pump-thaw cycles and purged with argon for 30 min. Polymerization was carried out at 65°C for 14 hrs. The polymer was isolated by precipitation from cold hexane (50 mL) and lyophilized to obtain a light-yellow solid in 77 wt% yield. ^1^H NMR (400 MHz, CDCl_3_): DP = 85%. GPC: 0.1 M NaNO_3_ at pH 2.6 (CH_3_CO_2_H); *M_n_* = 20,748 gmol^−1^, *Đ* = 1.161, calculated *dn*/*dC* = 0.198.

For the GPC analysis, the stationary phase consisted of a Tosoh TSKgel G5000 cationic aqueous solvent column (300 mm length, 7.8 mm ID) and a Tosoh TSKgel G3000 cationic aqueous solvent column (300 mm length, 7.8 mm ID) connected in series. The two chromatography columns were preceded by TSKgel PWXL guard column (40 mm length, 6 mm ID). Isocratic elution was performed with a flow rate of 0.5 mL/min, and 50 μL of sample at a concentration of 3 mg/mL was injected.


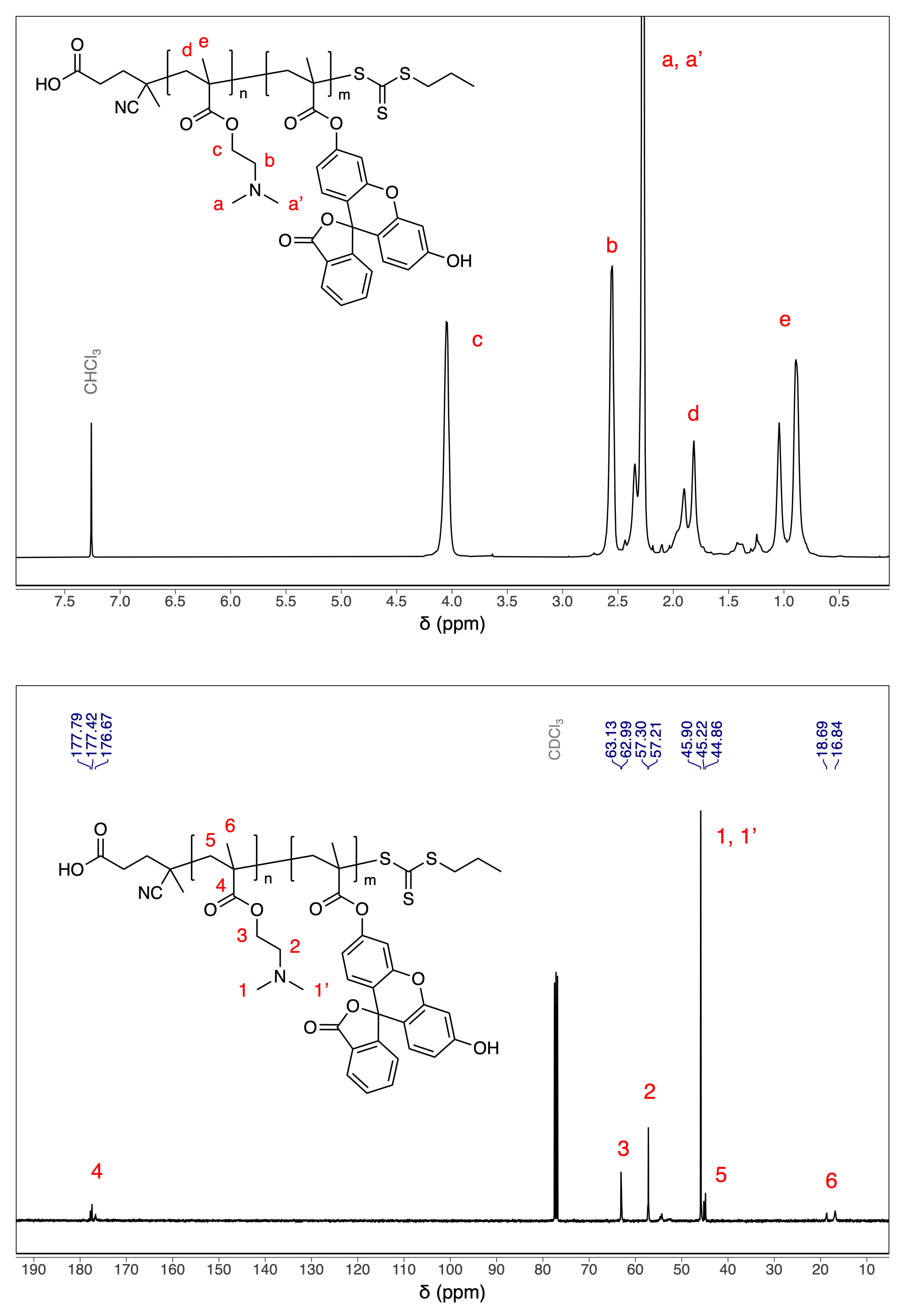


**Fig. S4.** Top: ^1^H NMR spectrum of FITC-PDMAEMA acquired in CDCl_3_ and referenced against residual CHCl_3_. Due to small amount of FITC present, no signals were observed in the aromatic region. Bottom: proton decoupled ^13^C NMR spectrum of FITC-PDMAEMA acquired in CDCl_3_ and referenced against residual CDCl_3_.


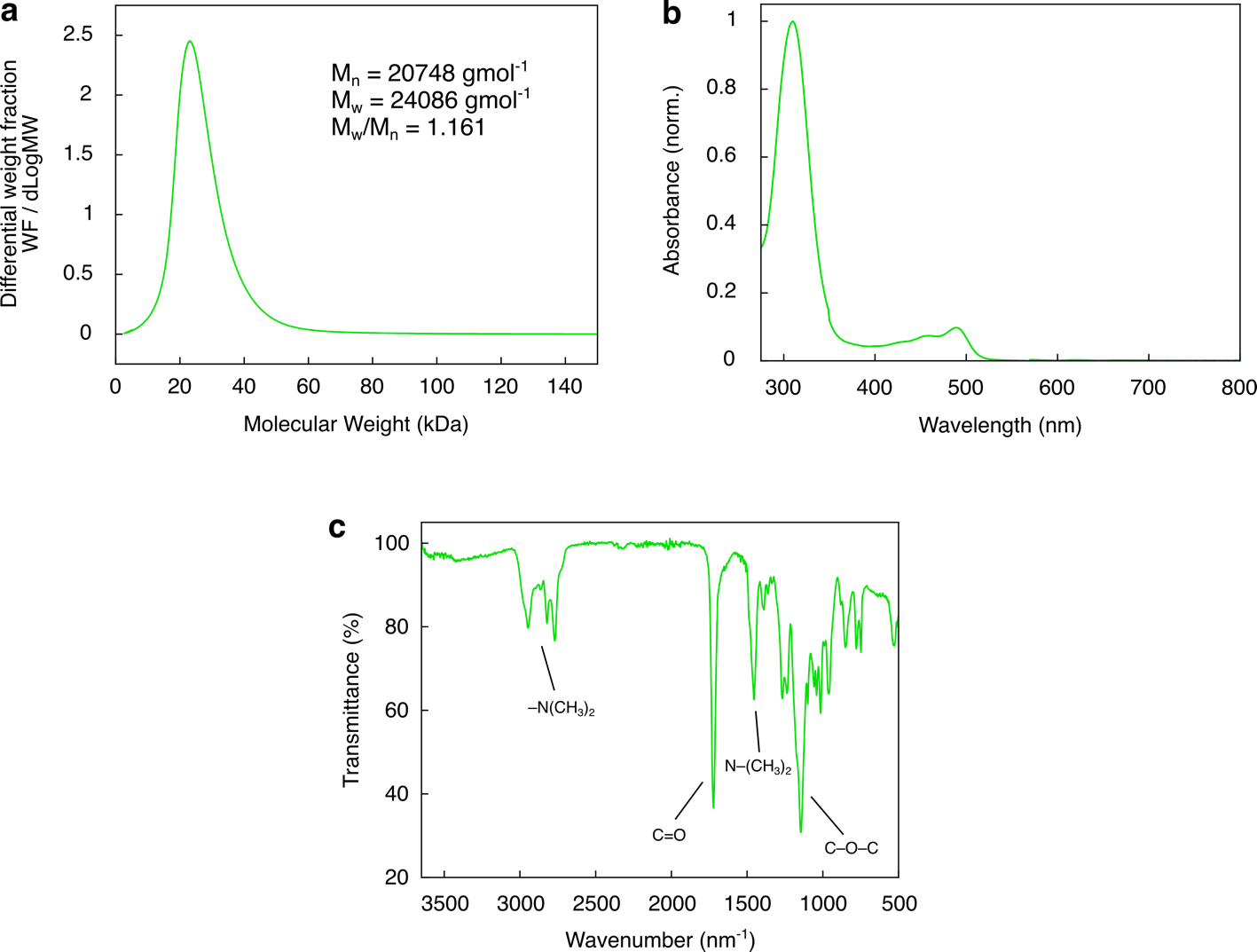


**Fig. S5.** Characterization of FITC-PDMAEMA. **a,** GPC molar mass distribution. **b,** Normalized UV-vis spectrum acquired in Milli-Q water at a concentration of 1 mg mL^-1^. In contrast with the UV-vis spectrum of PDMAEMA, the UV-vis spectrum of FITC-PDMAEMA shows clear presence of FITC. The degree of labelling was 0.02, meaning that not all polymer chains contain an FITC moiety. This degree of labelling was sufficient for all our experiments. **c,** ATR-FT-IR spectra of FITC-PDMAEMA.

- 1. **Synthesis of poly(acrylic acid)-co-rhodamine B methacrylate sodium salt (RITC-PAA)**

Acrylic acid (0.499 g, 6.92 mmol), methacryloxyethyl thiocarbamoyl rhodamine B (0.93 mg, 1.4 μmol), ACVA (2.24 mg, 8.00 μmol) and CPP (23.5 mg, 80.0 μmol) were dissolved in 1 mL of 1,4-dioxane in a Schlenk tube equipped with a stirrer bar. DMF (0.1 mL) was added as an internal standard to calculate the degree of polymerization. The tube was sealed, the solution was degassed with three freeze-pump-thaw cycles and purged with argon for 30 min. Polymerization was carried out at 70°C for 2 hrs. The polymer was isolated by precipitation from cold diethyl ether (50 mL). To form the sodium salt, after elimination of the solvent, the polymer has been dissolved in Milli-Q water and the pH has been adjusted to 8.5 by adding NaOH 0.5 M aliquots. The resulting polymer has been dialyzed against water and lyophilized to obtain a pink solid in 20 wt% total yield. ^1^H NMR (400 MHz, D_2_O): DP = 36%, calculated molecular weight = 3,208 g mol^-1^. GPC: 0.15 M PBS; *M_n_* = 4,358 gmol^−1^, *Đ* = 1.068, calculated *dn*/*dC* = 0.159.

For the GPC analysis, the stationary phase consisted of two Malvern A6000M aqueous solvent columns (300 mm length, 8 mm ID) and an A2500 column (300 mm length, 8 mm ID) connected in series. Isocratic elution was performed with a flow rate of 0.6 mL/min, and 150 μL of sample at a concentration of 4 mg/mL was injected.


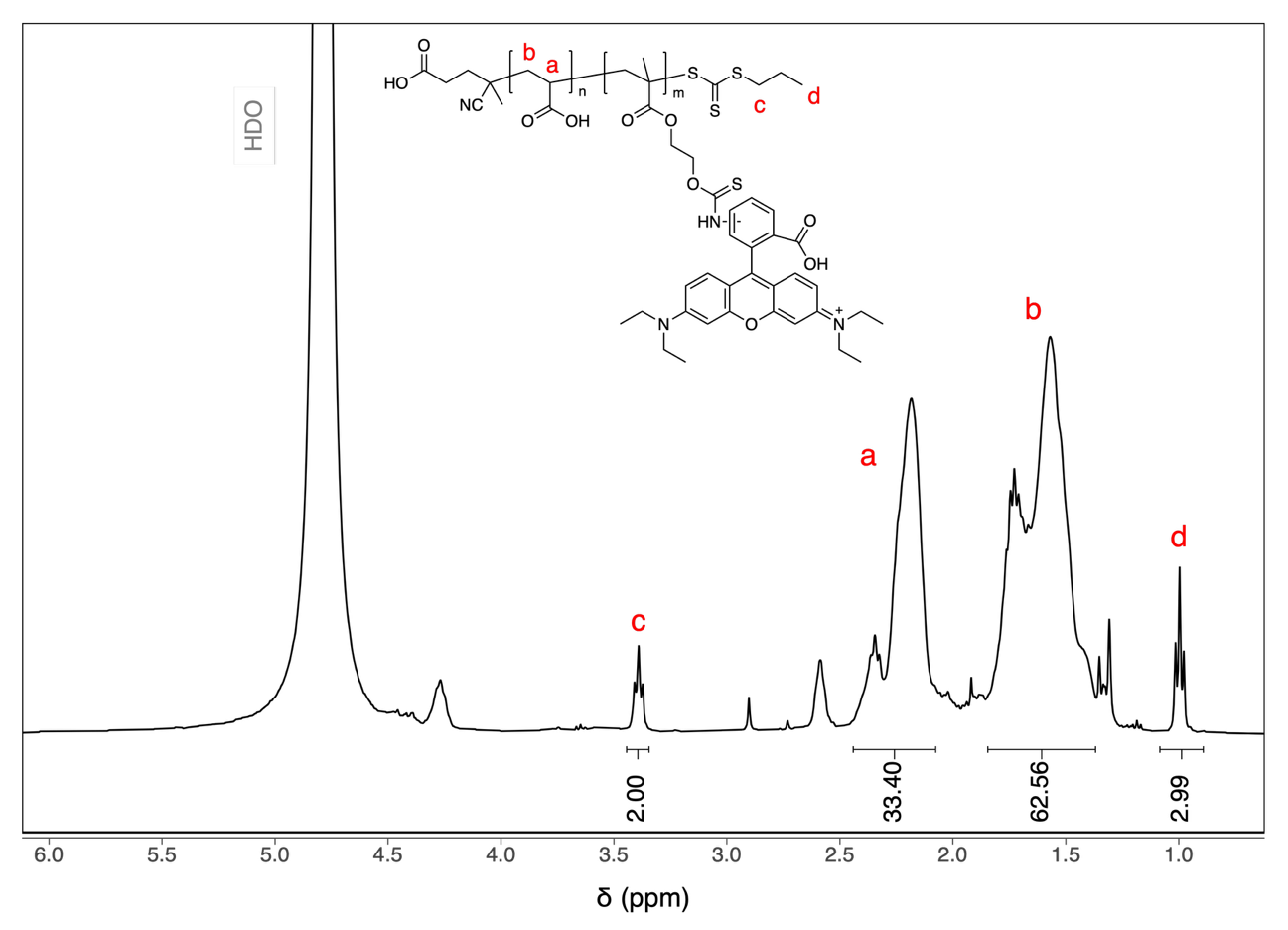


**Fig. S6.** ^1^H NMR spectrum of RITC-PAA acquired in D_2_O and referenced against residual HDO. Peaks corresponding to the CPP RAFT agent (c, d) were integrated to calculate the polymer molecular weight. The ^13^C NMR spectrum could not be obtained due to two factors: the polymer's poor solubility in common deuterated organic solvents, and the absence of detectable signals in D_2_O. The latter was likely caused by rapid ion-exchange dynamics and broadening effects typical of polyelectrolytes.


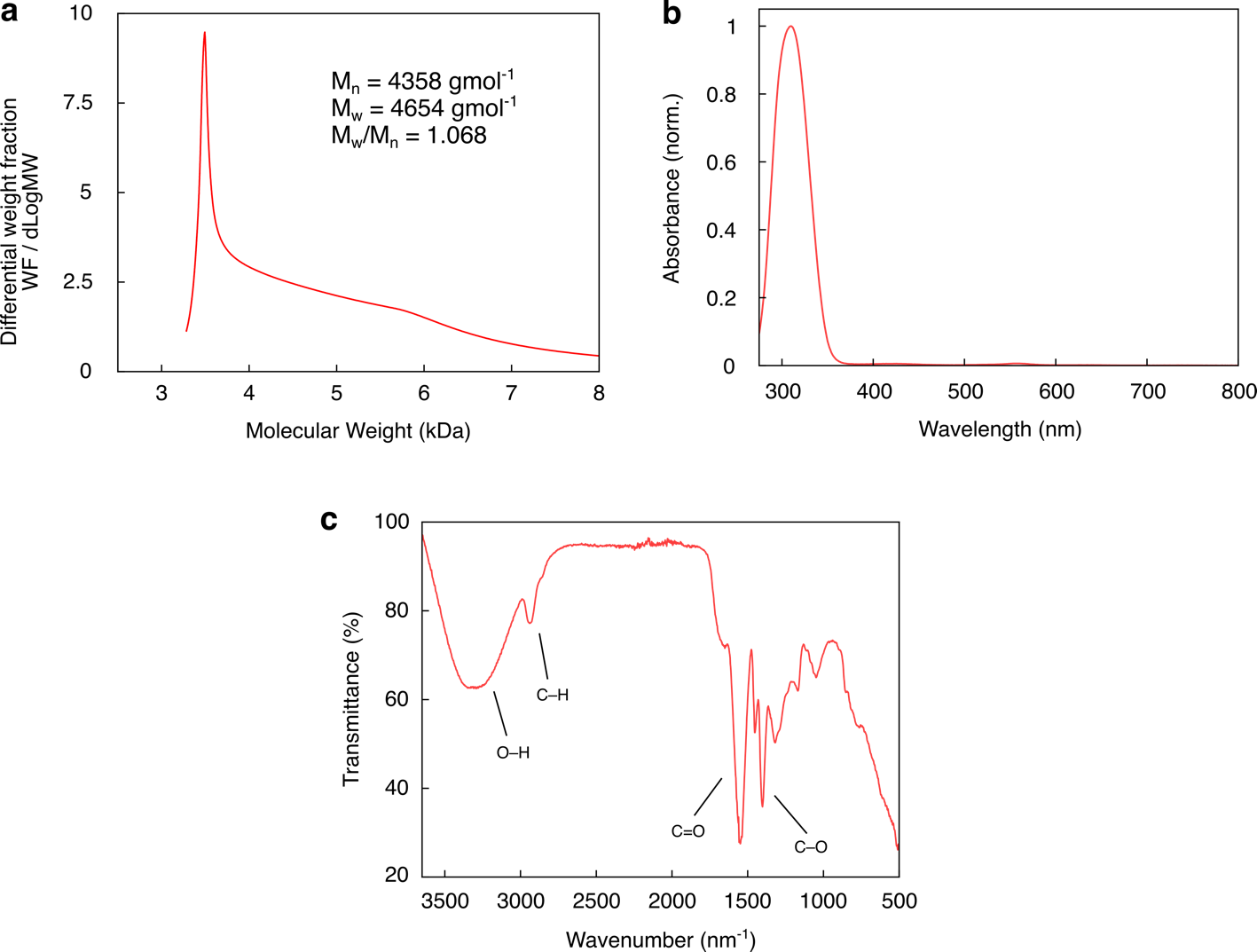


**Fig. S7.** Characterization of RITC-PAA. **a,** GPC molar mass distribution. **b,** Normalized UV-vis spectrum acquired in Milli-Q water at a concentration of 1 mg mL^-1^. The UV-vis spectrum shows presence of RITC. The degree of labelling was 0.0004, meaning that not all polymer chains contain an RITC moiety. This degree of labelling was sufficient for all our experiments. **c,** ATR-FT-IR spectra of RITC-PAA.

1. Generation of phase diagrams

To obtain the phase diagram for the different pairs of polycations and polyanions, the concentration of the starting solution for each polymer was fixed at 5 mM, 10 mM, 20 mM, or 50 mM, the pH was adjusted to 6.5, and the mixing ratio between polycation and polyanion was systematically varied. The final volume of the solution was always 400 µL. To fabricate the coacervate vesicles, the general procedure outlined in the methods section was followed. The formation of the coacervate vesicles was confirmed *via* optical microscopy both immediately after shaking and 5 minutes later. To further assess the self-assembly of the vesicles, the samples were also analyzed using cross-polarized microscopy. Properly formed coacervate vesicles showed a characteristic birefringence.


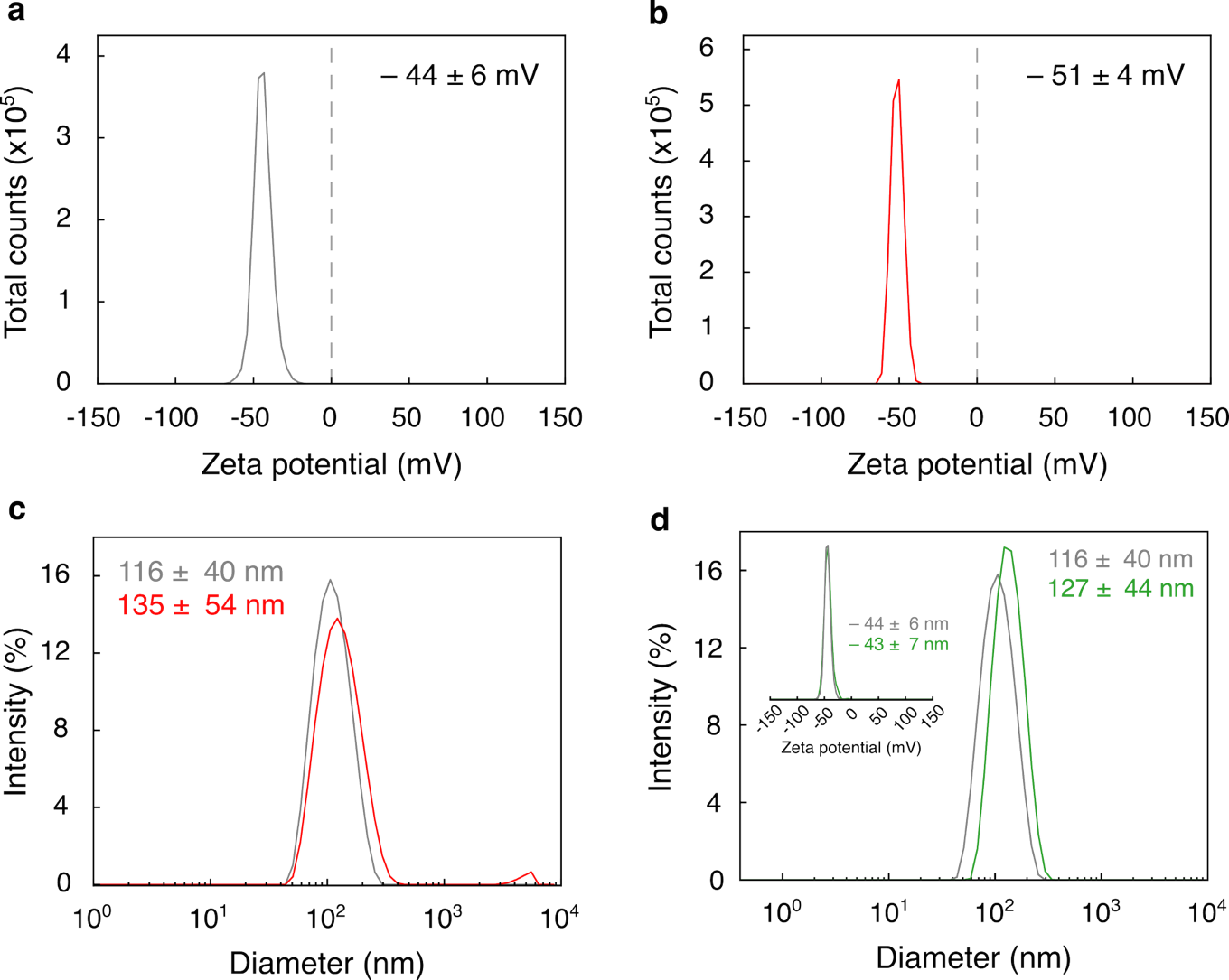


**Fig. S8. a,** Zeta potential measurement for the 1:4 PDMAEMA/PAA (10 mM) molar ratio mixture prior to shaking, showing an initial potential of −44 ± 6 mV. **b,** Zeta potential measurement for the same 1:4 PDMAEMA/PAA (10 mM) mixture after 30 s of manual shaking, with a slight decrease in potential to −51 ± 4 mV, indicating an increase in negative surface charge after agitation. **c,** Size distribution profiles of the 1:4 PDMAEMA/PAA (10 mM) mixture before (grey plot) and after (red plot) manual shaking. After shaking, DLS continues to show the presence of nanocoacervates, and a small peak appears in the micrometer size range indicating formation of much larger structures. **d,** Size distribution profiles of 1:4 PDMAEMA/PAA (10 mM) nanocoacervates, freshly prepared (grey plot) and after 7 days left undisturbed (green plot). DLS analysis shows that nano-coacervates persist with comparable diameters after one week. The inset reports their corresponding normalized zeta potential analysis, indicating no significant changes. These results confirm that nanocoacervates are stable over long time, with their strong negative charge preventing coalescence.


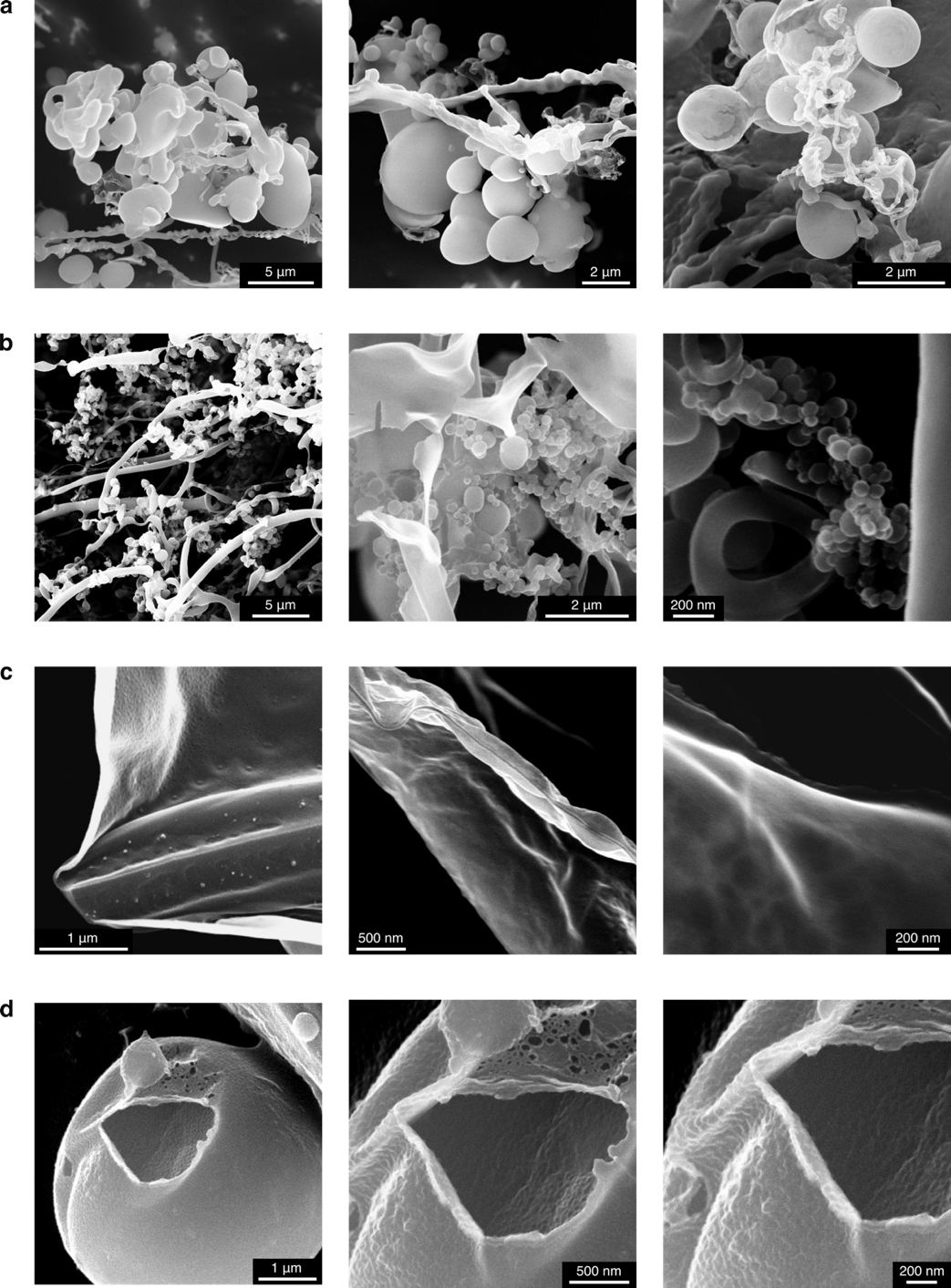


**Fig. S9.** Representative Cryo-SEM images of the PDMAEMA/PAA system at various molar ratios after sample sublimation. **a,** Coacervate microdroplets (>1 µm in diameter) were observed at a 1:1 PDMAEMA/PAA (10 mM) molar ratio. **b,** Numerous distinct nanocoacervates (*ca.* 100 nm in diameter) are observed were observed at a 1:4 PDAMEMA/PAA (10 mM) molar ratio and before manual shaking. **c,** Membrane details of coacervate vesicles formed from a 1:4 PDMAEMA/PAA (10 mM) molar ratio after 30 s of manual shaking. **d,** Detailed Cryo-SEM images of a single coacervate vesicle. These images reveal a rough vesicle surface resulting from the fusion of nanocoacervates *ca.* 100 nm in diameter, providing evidence that mechanical energy can promote the adhesion of individual nanodroplets into a microcompartmentalized structure. Elongated structures (diameter *ca.* 1 µm, length > 5 µm) visible in (**a**) and (**b**) arise from sample preparation artifacts, attributed to sublimation effects of dissolved polymers in the aqueous phase during freezing. These structures are confirmed as artifacts by their absence in both DLS and SANS measurements.

1. Small-angle neutron scattering (SANS) experiments

Samples for SANS analysis were prepared by mixing the appropriate volumes of 10 mM solutions of PDMAEMA and PAA in D_2_O. These solutions were filtered through 0.22 μm nylon filters (Artiglass). The pD (pH in D_2_O) was adjusted to 6.5 using 0.5 M DCl, and measurements were conducted using a digital pH-meter equipped with a glass electrode. pH-meter readings in D_2_O were corrected using the formula pD = pH + 0.40.^2^

Samples were studied both before and after 30 s of manual shaking and prepared by directly mixing the solutions of the two polymers in a 1.75 mL glass vial. Vesicle samples were prepared from the resulting solutions, which were vigorously shaken by hand for 30, as described in the methods section. The solutions were then transferred to Hellma quartz spectrophotometer cuvettes with a path length of 2 mm for SANS measurements. The raw scattering data were reduced using Mantid software, where the scattering from the solvent (D_2_O) and the empty cell was subtracted.

For data analysis in SasView, the scattering profiles of the coacervate microdroplets samples were fitted using a correlation length model, as previously reported, to determine the overlapping of polymer chains in the complex coacervate phase.^3^ The scattering intensity *I(q)* is calculated as:

$I\left( q \right)= \frac{A}{q^{n}}+\frac{C}{{1+(q\xi)}^{m}}+background$ (**Supplementary Equation 1)**

where the two multiplicative factors 𝐴 and 𝐶, and the two exponents 𝑛 (Porod) and 𝑚 (Lorentz) are used as fitting parameters. The *x* parameter is a correlation length for the polymer chains. The typical correlation length ξ of complex coacervates is a few nanometers, indicating polymer chain overlap.

**Table S1:** SANS fitting parameters for 1:1 PDMAEMA/PAA (10 mM) molar ratio coacervate microdroplets before (a) and after application of impulsive force (b), and for 10 mM solution of PDMAEMA in D_2_O.

|  | *A* | *C* | *backgr* (10^3^) | ***ξ* (nm)** | *n* | *m* | *χ²* |
| --- | --- | --- | --- | --- | --- | --- | --- |
| **a** | 5.55 ∙ 10^-12^ | 0.08 | 1.10 | 2.17 ± 0.04 | 4.00 | 2.37 | 0.70 |
| **b** | 5.70 ∙ 10^-8^ | 0.07 | 1.93 | 2.55 ± 0.06 | 3.05 | 2.17 | 0.78 |
| **PDMAEMA** | 2.32 ∙ 10^-10^ | 0.08 | 3.62 | 2.12 ± 0.04 | 3.57 | 2.21 | 1.49 |

For the 1:4 molar ratio PDMAEMA/PAA samples, a core-shell ellipsoid model was applied to fit the data.

The intensity is given by:

$I\left( q,\alpha\right)=\frac{scale}{V}F^{2}\left( q,\alpha\right)+background$ **(Supplementary Equation 2)**

where *α* is the orientation angle of the ellipsoid, and where:

$F\left( q,\alpha\right)=f\left( q,R_{e},R_{e}\cdot x_{core},\alpha\right)+f\left( q,R_{e}+t_{shell},R_{e}{\cdot x}_{core}{+ t}_{shell}\cdot x_{polarshell},\alpha\right)$ **(Supplementary Equation 3)**

And:

$f\left( q,R_{e},R_{p},\alpha\right)=\frac{3\Delta\rho V(\sin\left( qr \right)-qr cos(qr)}{(qr)^{3}}$ **(Supplementary Equation 4)**

For:

$r=[R_{e}^{2}{sin}^{2}\alpha+ R_{p}^{2}{cos}^{2}\alpha]^{1/2}$ **(Supplementary Equation 5)**

where *V* is the volume of the ellipsoid with *R_e_* as the equatorial radius, and *R_p_ = R_e_* ∙ *x_core_* as the polar radius. *t_shell_* is the thickness of the shell at the equator, and *∆ρ* is the scattering length density difference, *i.e.*, *∆ρ* = (*ρ_shell_ – ρ_solvent_*) *or* (*ρ_core_ – ρ_solvent_*).

**Table S2:** SANS fitting parameters for the 1:4 PDMAEMA/PAA (10 mM) molar ratio nanocoacervates before (a) and after application of impulsive force (b).

|  | *Scale* | *backgr* (10^3^) | ***R_e_* (nm)** | ***x_core_*** | ***t_shell_* (nm)** | *ρ_shell_*  *(10^6^ Å^-2^)* | *ρ_core_*  *(10^6^ Å^-2^)* | *χ²* |
| --- | --- | --- | --- | --- | --- | --- | --- | --- |
| **a** | 0.10 | 3.77 | 35.9 ± 4.6 | 0.75 ± 0.13 | 61.8 ± 3.5 | 6.33 | 5.84 | 1.02 |
| **b** | 0.12 | 1.80 | 48.0 ± 1.6 | 0.53 ± 0.02 | 57.2 ± 2.9 | 6.33 | 5.84 | 0.88 |

Fitting parameters were optimized to achieve the best agreement between the models and the experimental data.


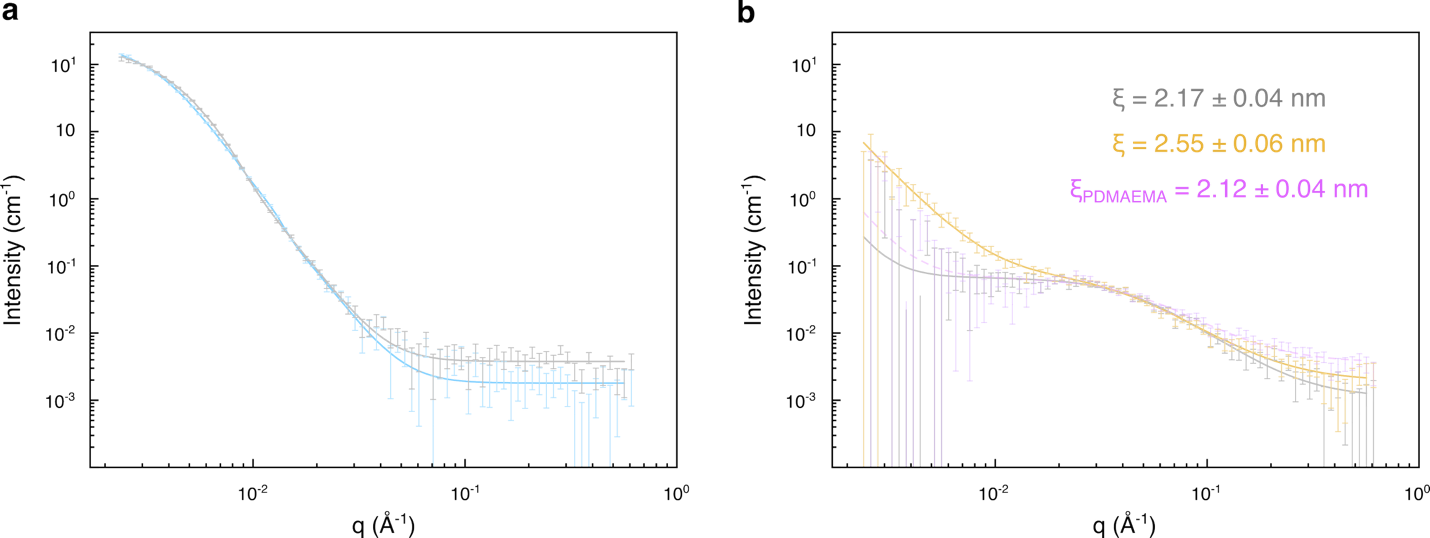


**Fig. S10.** SANS analysis of PDMAEMA/PAA (10 mM) mixtures in D₂O at varying polymer molar ratios. Samples were prepared as described in the methods section. Each graph shows the scattering data before (grey curve and error bars) and after (colored curve and error bars) the application of an impulsive force. **a,** SANS data of nanocoacervates formed at a 1:4 PDMAEMA/PAA (10 mM) molar ratio: after manual shaking, the SANS curve remains practically unchanged, with small changes in the radius of the core and the thickness of the shell of the nanocoacervates. **b,** SANS data of coacervate microdroplets formed at a 1:1 PDMAEMA/PAA (10 mM) molar ratio: data were fitted using a correlation length model as previously reported for similar systems. The correlation length value slightly decreased from *ξ* = 2.17 ± 0.04 nm to *ξ* = 2.55 ± 0.06 nm upon shaking. The upward shift in the low-q region post-shaking suggests clustering and the formation of larger coacervate droplets. In general, these results are comparable with those obtained from PDMAEMA semi-diluted solution in D_2_O, which is reported in the graph as a pink dashed curve. In this latter case, the correlation length value is *ξ* = 2.12 ± 0.04 nm.

1. Preparation of coacervate vesicles

Stock solutions of each polymer were prepared in Milli-Q water. For molarity calculations, we referred to the molecular weights (Mw) of the monomers (DMAEMA: 157.21 g mol^-1^, AA(Na salt): 94.05 g mol^-1^, DDA chloride: 161.67 g mol^-1^, average nucleobase: 303.70 g mol^-1^, PLys: 223.12 g mol^-1^). The pH of the solutions was adjusted to 6.5 by the addition of either HCl (0.5 M) or NaOH (0.5 M).

For the preparation of coacervate vesicles using a 1:4 PDMAEMA/PAA molar, 100 μL of 10 mM PDMAEMA stock solution and 400 μL of 10 mM PAA stock solution were combined in a 1.75 mL glass vial. The solution looked clear. In order to form the coacervate vesicles, the resulting solution was vigorously shaken by hand for 30 s with a hand gesture that is identical to that used to lower the temperature of a thermometer, thereby producing strong shockwaves in the liquid. For instance, a vigorous stirring with a stirrer-bar or sonication failed to produce coacervate vesicles, whereas vortexing (which combines shear and impact forces) generated only a negligible number of coacervate vesicles (see **Fig. S11**).

The same method was used to generate all the different types of coacervate vesicles described in this work, with the only difference that the proper polymer solutions and/or molar ratios were used instead of a 1:4 PDMAEMA/PAA (10 mM) molar ratio.

All microscopy images were acquired from vesicles settled on the glass surface. Upon contact with glass, coacervate vesicles maintain their structural integrity, in contrast to coacervate microdroplets, which rapidly wet the glass surface and lose their spherical morphology.


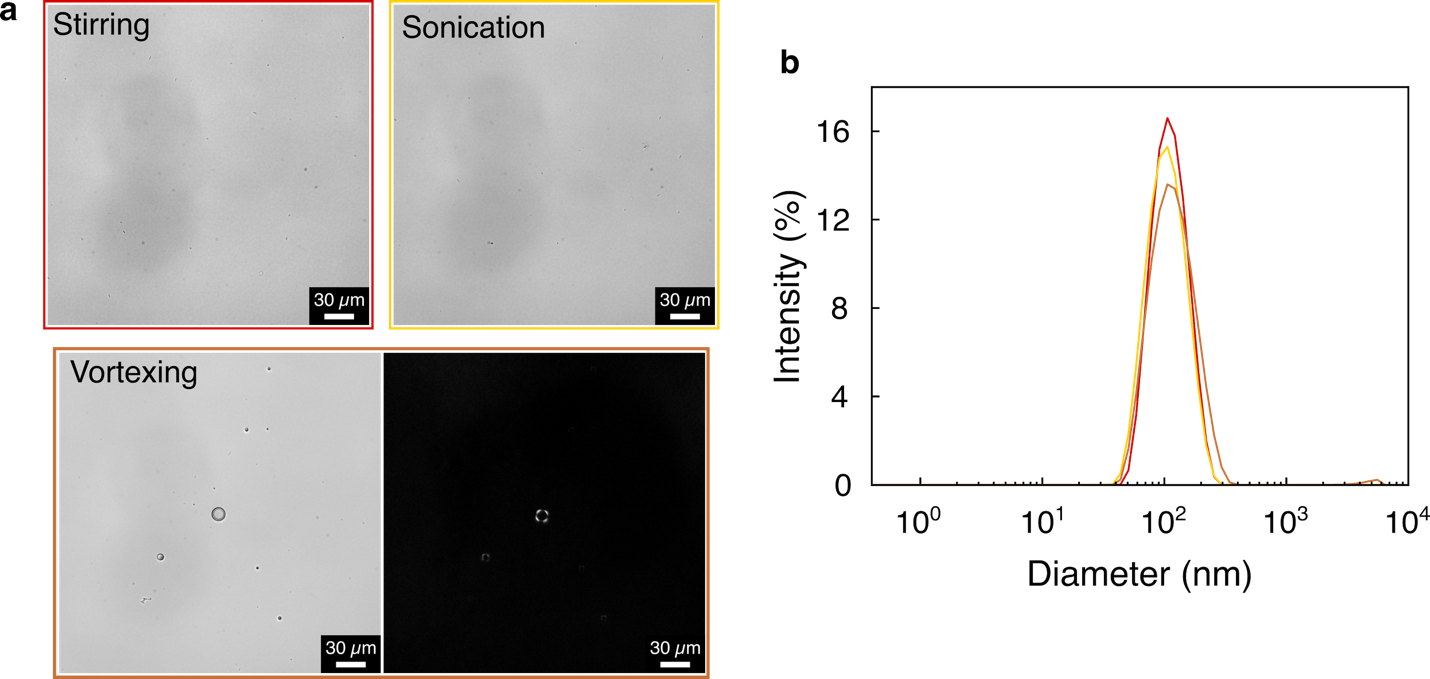


**Fig. S11.** **a,** Microscopy and **b,** DLS analysis of 1:4 PDMAEMA/PAA (10 mM) molar ratio mixture after being subjected to various forms of mechanical energy for 30 s. Stirring (2000 rpm) and sonication (red and yellow color code, respectively) did not produce vesicles and caused no detectable changes in the nanometric properties of the coacervate droplets, as confirmed by the DLS size distribution. By contrast, vortexing (brown color code), which combines shear and impulsive force, led to the formation of a small number of birefringent vesicles, as confirmed by both microscopy and DLS (appearance of a small peak in the micrometer size range). These results further support that impulsive force, rather than shear, is required for coacervate vesicle formation.


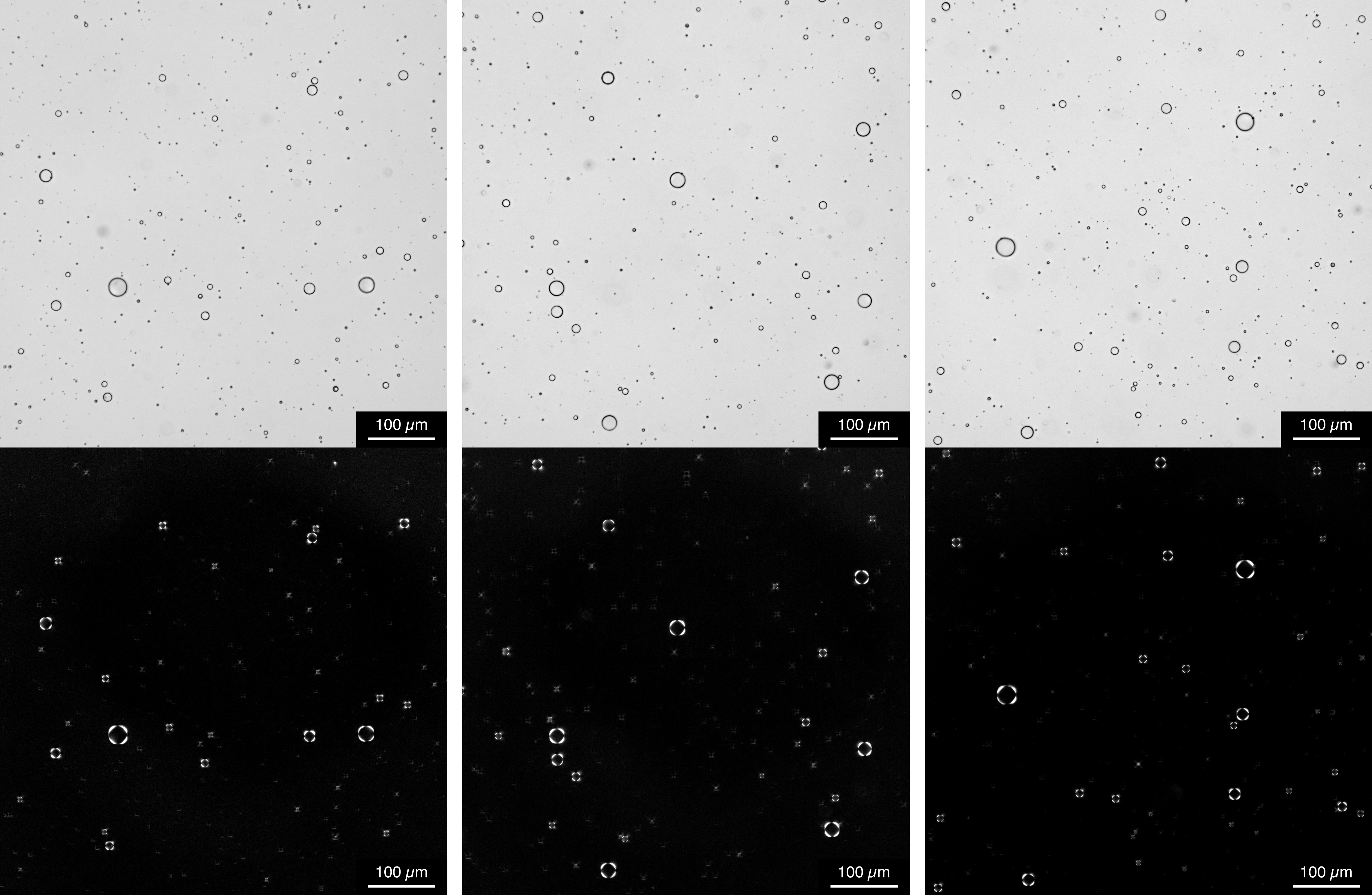


**Fig. S12.** Gallery of representative microscopy images for the size distribution study of a population of a 1:4 PDMAEMA/PAA (10 mM) molar ratio coacervate vesicles. Each microscopy image was analyzed using both high resolution brightfield microscopy and polarized light microscopy. The study was conducted on three independent samples, prepared as described in the methods section, with multiple representative images captured across each sample using a 20× objective lens. Only coacervate vesicles exhibiting a Maltese-cross pattern under cross-polarized light, indicative of a highly ordered membrane structure, were included in the analysis.

1. Reversible generation of coacervate vesicles

To study the disassembly and the regeneration of the coacervate vesicles, coacervate vesicles were prepared as described in the methods section, and were subsequently monitored over time at room temperature using brightfield microscopy. The size and count of vesicles and coacervate microdroplets were automatically measured and analyzed using the built-in ZEN Blue software on the ZEISS Axio Observer microscope. This software enabled the precise determination of size distribution, and the quantification of vesicle count at each time point.

To investigate the reversible formation-disassembly process of coacervate vesicles, the same methodology was followed, with the only difference that the disassembly process was accelerated using a ThermoFisher tube revolver in agitation mode. After complete dissolution (24 hrs), samples were re-shaken manually for 30 s to reform the coacervate vesicles, and the analysis process using brightfield microscopy was repeated.


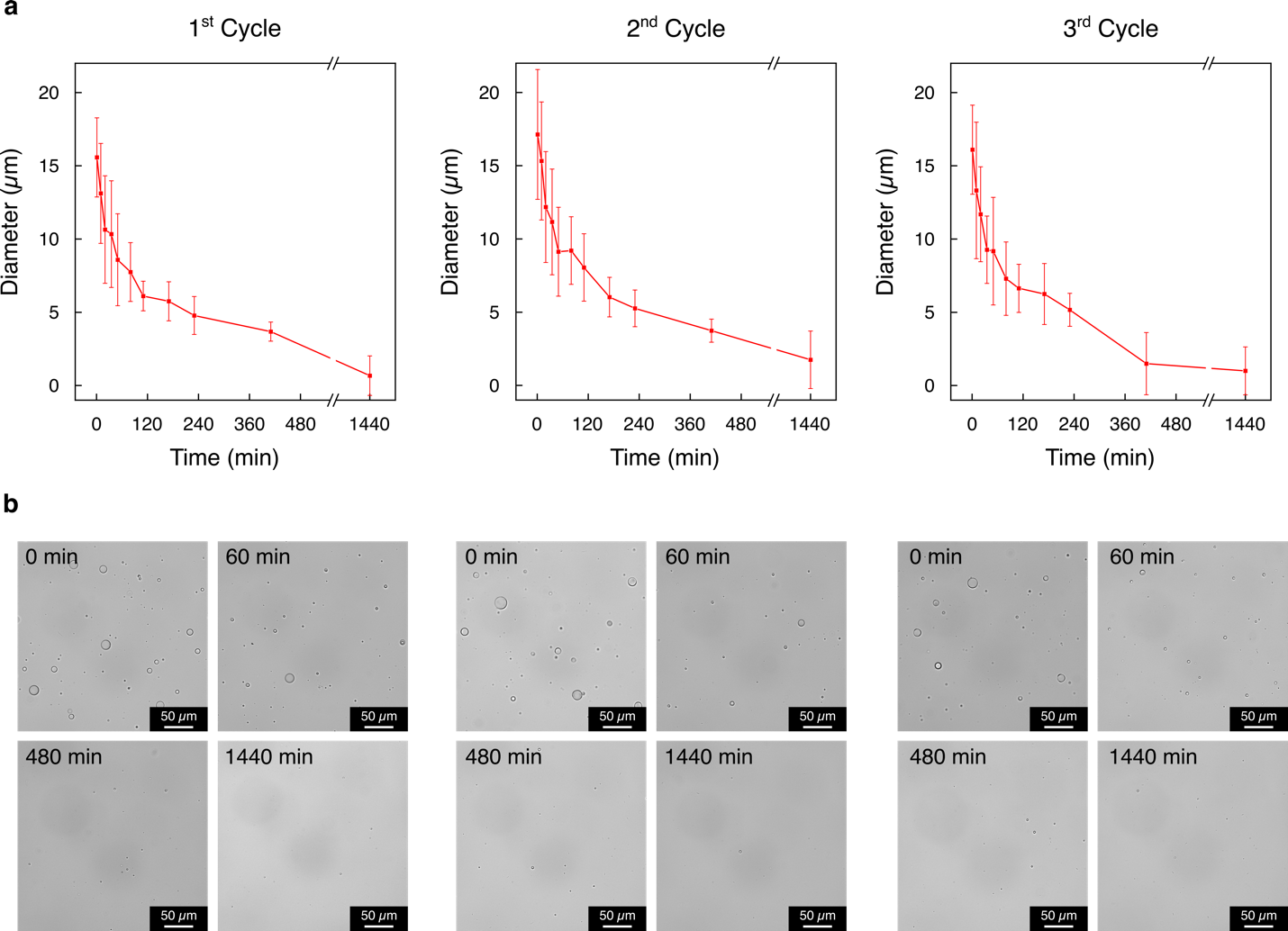


**Fig. S13.** Reversible formation of coacervate vesicles from a 1:4 molar ratio mixture of PDMAEMA/PAA (10 mM). **a,** Progressive decrease in coacervate vesicle diameter over a 24-hour period (1440 minutes). To accelerate the dissolution process, samples were kept under constant agitation using a tube revolver throughout the entire duration of the experiment. Observations began immediately after manual shaking (t = 0 min) and continued at regular intervals. The 2^nd^ and 3^rd^ cycles represent the same vesicle system, which was manually shaken again after a period of 24 and 48 hrs, respectively. In each of these cycles, a quasi-exponential decay in vesicle diameter is observed, with vesicle size initially averaging around 15 µm and decreasing gradually to complete disassembly. Error bars = standard deviation (n=25). **b,** Representative brightfield microscopy images at selected time points (0, 60, 480, and 1440 minutes) for each of the three cycles. These images clearly show the reduction in vesicle size, alongside the gradual dissolution process observed over time in each cycle. This visual sequence is consistent with the quantitative trends presented in panel **a**, capturing the transition from initial vesicle formation to complete disassembly.


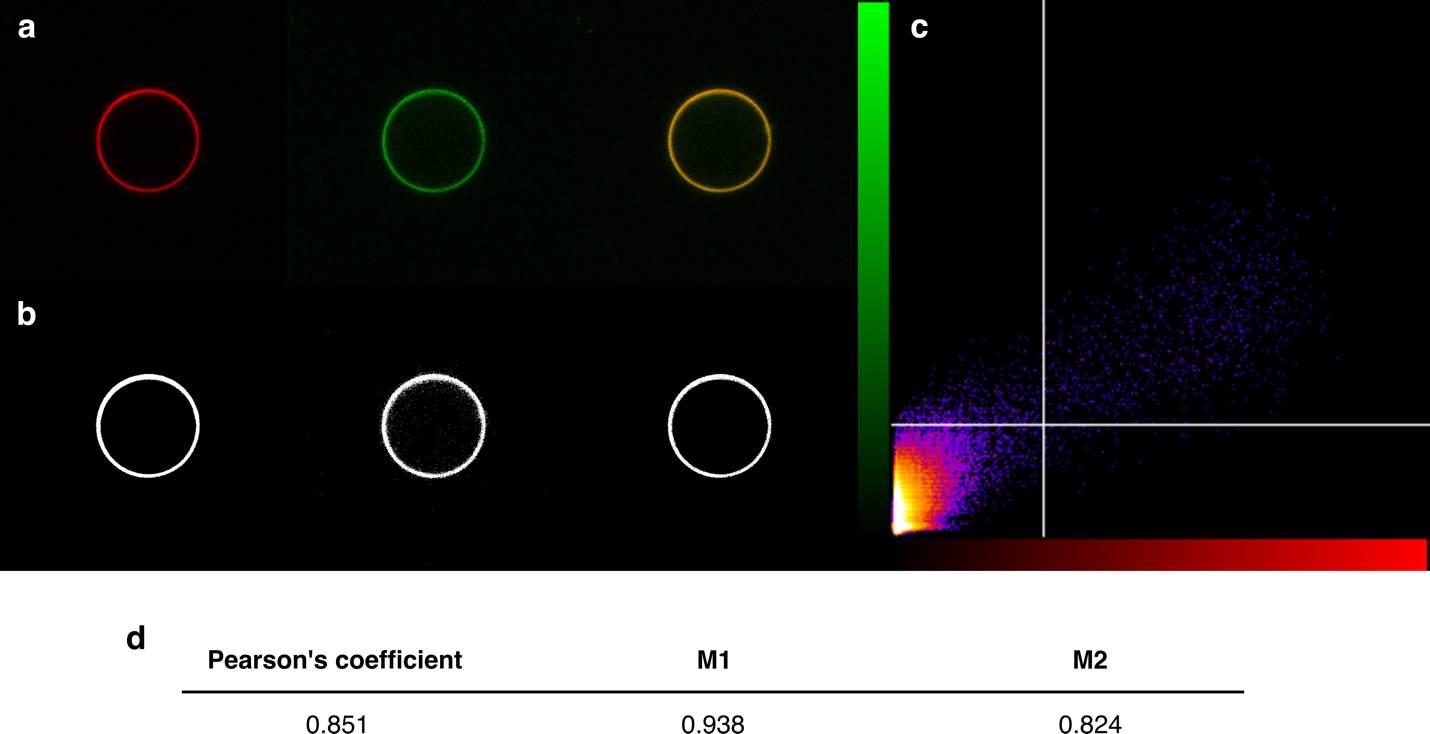


**Fig. S14.** Colocalization studies of coacervate vesicles formed from fluorescent polymers. Coacervate vesicles were prepared at a 1:4 PDMAEMA/PAA (10 mM) molar ratio, each 10 mM polymer stock solution contained a 10 mol% of the corresponding fluorescently tagged polymer, FITC-PDMAEMA or RITC-PAA. **a,** Red, green, and merged channels of a confocal laser scanning microscopy image of a single representative coacervate vesicle. **b,** Binary thresholded images of the red, green, and merged channels showing pixel overlap. **c,** Colocalization scatterplot of the individual pixels from the paired images, with white lines indicating threshold levels for both channels. Most pixels above the threshold values are aligned in the top-right quadrant, suggesting nearly complete colocalization. **d,** Pearson’s correlation coefficient and Mander’s coefficients (M1, M2) for FITC-PDMAEMA and RITC-PAA colocalization. Both Pearson’s and Mander’s coefficient values approach 1, indicating that nearly all pixels of the two thresholded channels overlap, demonstrating high colocalization of the two polymers. Colocalization parameters were quantified using the BIOP JACoP plugin in ImageJ, applying an automatic threshold set by the Otsu method.

1. Fluorescence recovery after photobleaching (FRAP) experiments

Coacervate vesicles were formed utilizing the general procedure described in the methods section, with the only difference that FITC-PDMAEMA was used in place of PDMAEMA. Coacervate vesicles were formed at a 1:6 molar ratio between FITC-PDMAEMA and PAA (10 mM), whereas complex coacervate microdroplets were formed at a molar ratio of 1:1. The two samples were used to perform FRAP experiments on an Olympus FV3000 confocal laser scanning microscope utilizing a 60x oil immersion objective lens. For both samples excitation was performed with a 488 nm laser and the emission was recorded between 500 – 550 nm. Fluorescence recovery was recorded for 10 minutes with an acquisition rate of one frame every 30 s. To obtain a pre-bleaching reference, two images were taken before bleaching, always with an acquisition rate one frame every 30 s. Bleaching was performed using a 488 nm laser at 50% intensity for 2 s in a circular area of approximately 1 µm in radius, with a scanning speed of 2 µs/pixel. FRAP analysis was carried out using the latest version of ImageJ software. The fluorescence intensity in the selected area was normalized with the intensity in an area on the opposite side of the vesicle or microdroplet to account for any overall photobleaching effects, as shown in the formula:

$I_{norm}\left( t \right)= \frac{I_{bleach}\left( t \right)-I_{background}\left( t \right)}{I_{reference}\left( t \right)-I_{background}\left( t \right)}$ **(Supplementary Equation 3)**

Where, for every image at time of the experiment time lapse:

$I_{norm}\left( t \right)$ is the normalized fluorescence intensity.

$I_{bleach}\left( t \right)$ is the fluorescence intensity of the bleached region.

$I_{background}\left( t \right)$ is the fluorescence intensity of the background (outside the vesicle/microdroplet).

$I_{reference}\left( t \right)$ is the fluorescence intensity of the reference region (opposite side of the vesicle/microdroplet).


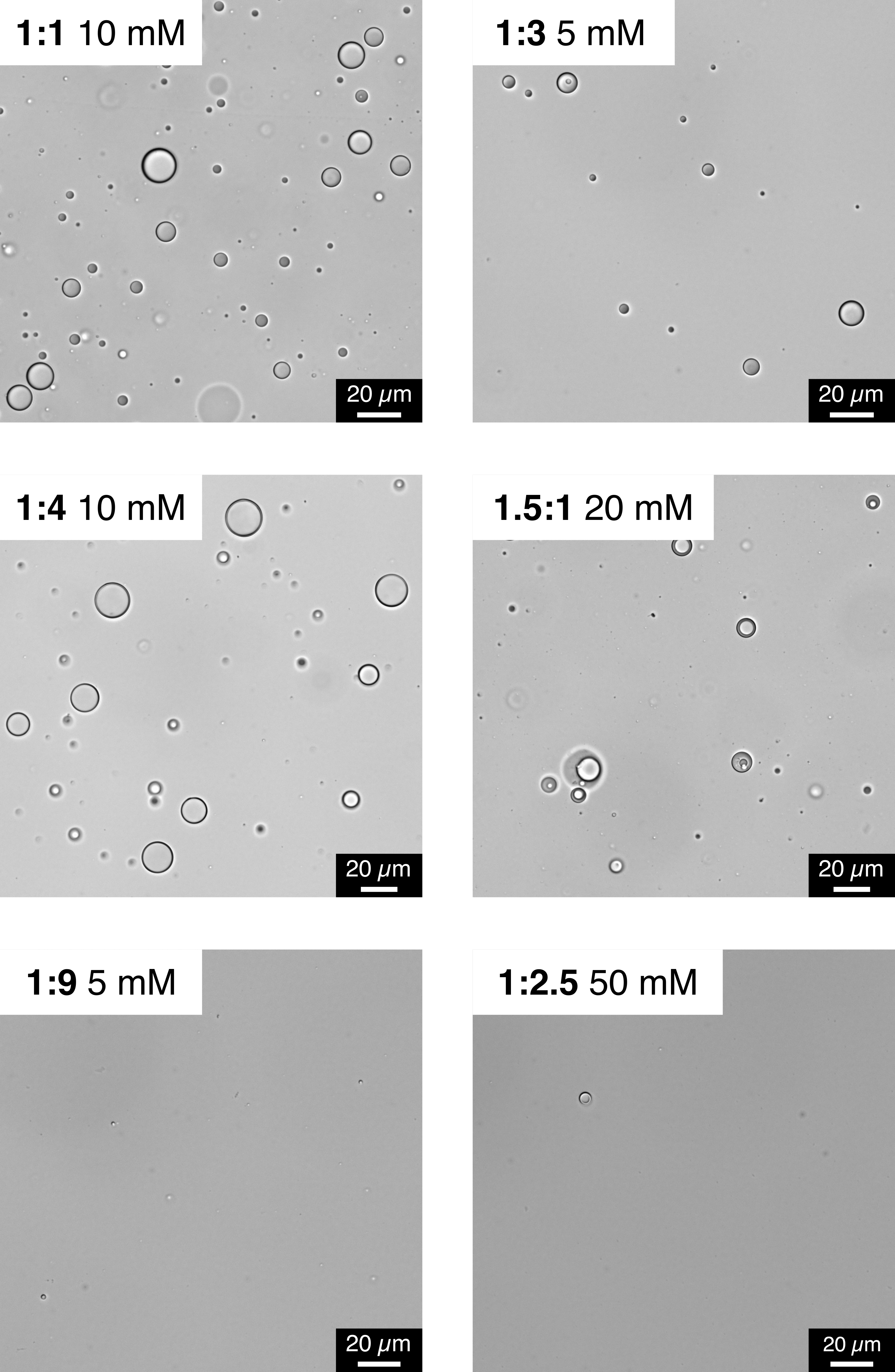


**Fig. S15.** Representative brightfield microscopy images of the binary PDMAEMA/PAA system referred to different regions of the phase diagram (Fig. 2A). For molar ratios within the yellow region of the phase diagram (1:1 and 1:3 PDMAEMA/PAA molar ratio), coacervate microdroplets were observed. Unlike the coacervate vesicles formed at a 1:4 PDMAEMA/PAA molar ratio, these microdroplets lacked an internal aqueous lumen, appeared fully dense, and rapidly coalesced or wetted the glass surface. The 1.5:1 PDMAEMA/PAA molar ratio system showed mixed behavior, with coacervate vesicles that disassembled more rapidly, resembling the dynamics of coacervate microdroplets. For molar ratios within the white region of the phase diagram (1:9 and 1:2.5 PDMAEMA/PAA molar ratio), no coacervation was observed.


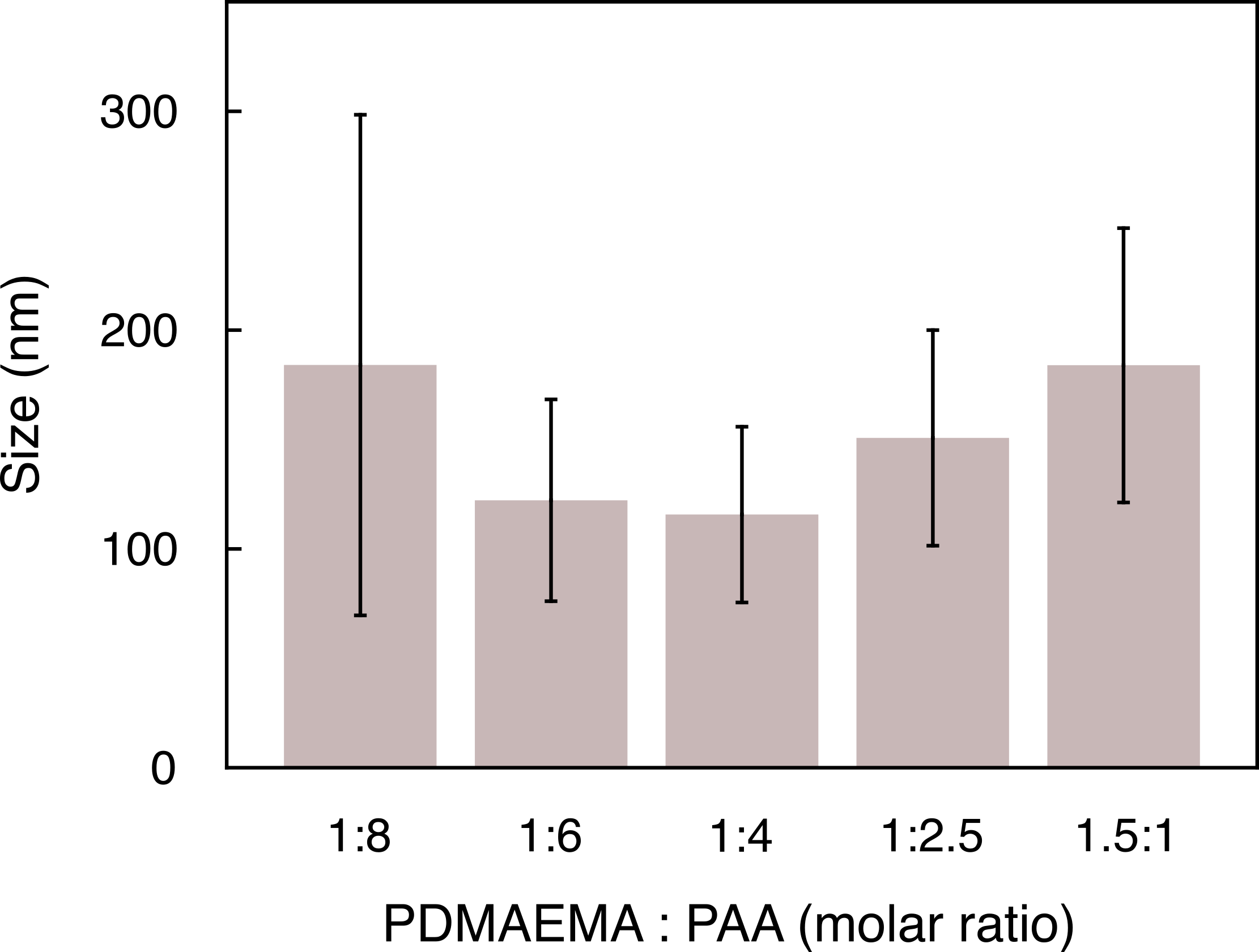


**Fig. S16.** DLS size distribution of nanocoacervates assembled from 10 mM PDMAEMA/PAA solutions at different molar ratios. Error bars indicate standard deviation across three measurements. Across all concentrations studied, nanodroplets 100-200 nm in diameter were detected. Reproducible size data at complex coacervate molar ratios (1:2, 1:1) could not be obtained due to rapid droplet coalescence and surface wetting.

1. Nanocoacervates fusion based on impulsive pressure

The formation of coacervate vesicles under mechanical agitation likely results from impact-induced fusion of nanodroplets, overcoming electrostatic and osmotic repulsion. This hypothesis is based on the fact that a jerking motion (*i.e.*, a sudden stop and reversal of movement) is required, suggesting that transiently high pressures are necessary.

At a 1:4 PDMAEMA:PAA molar ratio, the nanodroplets can be approximated as core-shell structures (see Figure 2d, Supplementary section 4, **Fig. S10**), where excess PAA forms a negatively charged shell with a measured zeta potential ζ = – 44.0 ± 6 mV. The electrical double-layer pressure stabilizing these droplets can be estimated using the Debye-Hückel approximation:

$P_{edl}\approx2\varepsilon_{0}\varepsilon_{r}\kappa^{2}\zeta^{2}\approx300-1000 \mathrm{Pa}$ **(Supplementary Equation 4)**

Where, $\varepsilon_{0}=8.85\times{10}^{-12} F m^{-1}$ the permittivity of the free space and $\varepsilon_{r}=78.5$ the dielectric constant of water, assuming the Debye constant $\kappa\sim\left( 1.25-1.80 \right)\times{10}^{7}m^{-1}.$^4,5^

Additionally, the osmotic pressure of the PAA shell, arising from confined monomers, can be estimated using the de Gennes blob scaling argument:^6^

$P_{osm}\approx\frac{k_{B}T}{s^{3}}$ **(Supplementary Equation 5)**

where s ~ 15 nm is the estimated mesh size of the PAA shell.^4^ This yields $P_{osm}\approx1200 \mathrm{Pa}$, giving a total repulsive pressure of:

$P_{rep}=P_{edl}+P_{osm}\approx1500-2200\mathrm{Pa}$ **(Supplementary Equation 6)**

To overcome this repulsion, the jerking motion applies an impact force to the liquid. For a 1 mL solution (~ 1 g) subjected to sudden motion reversal at *v* ~ 5 – 10 m s^-1^ over *Δt* ~ 50 – 100 ms (as estimated from video recordings of vial shaking during coacervate vesicle formation, see **Fig. S17**), the impulse-momentum equation gives:

$F_{i}\approx m\frac{2v}{\Delta t}\approx0.1-0.4 N$ **(Supplementary Equation 7)**

With an estimated impact area of A ~ 1 cm², the impact pressure is:

$P_{i}=\frac{F}{A} \approx1000-4000\mathrm{Pa}$ **(Supplementary Equation 8)**

Notably, in the context of natural phenomena, an impulsive pressure of this order of magnitude (*i.e.*, ~ 1000 – 2000 Pa) may be achieved by a falling rain drop of 2 mm in diameter reaching a ground impact velocity of 3 – 5 m s^-1^ and with an impact time of ~ 1 – 2 ms.^7^ The impulsive pressure exerted on rocks from crashing ocean waves could readily reach more than $10000 \mathrm{Pa}$, whilst it is plausible that a smaller pressure would be achieved by a gentler caressing wave.^8^

Since *P_i_* is comparable to *P_rep_*, the impact force likely overcomes nanodroplet electrostatic repulsion, triggering their fusion into vesicles. However, the vesicles gradually disassemble over time, suggesting that the fused structures exist in a metastable dissipative state rather than a thermodynamically favored one.


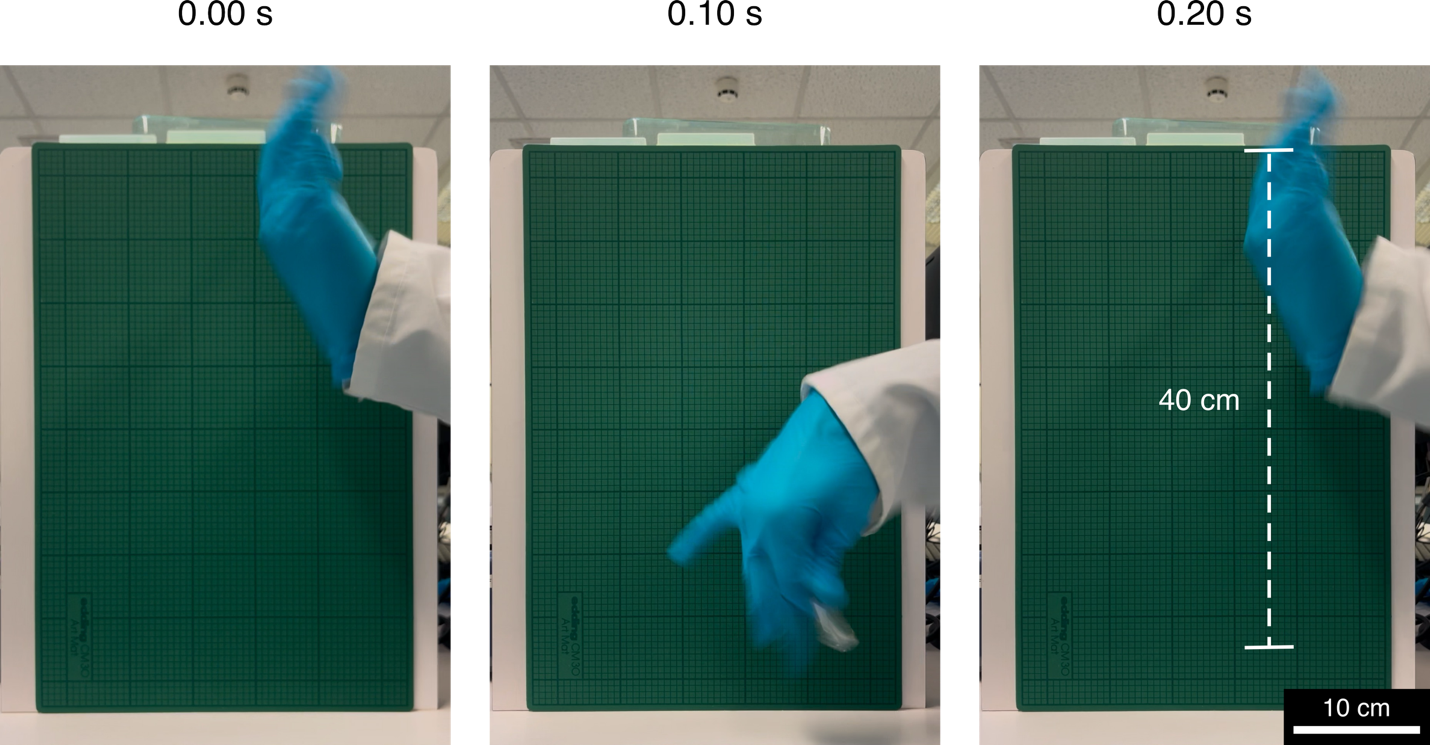


**Fig. S17.** Timeframes showing the manual shaking of a glass vial to produce coacervate vesicles. The amplitude of the movement was around 40-50 cm, with a frequency of 3-5 Hz.

1. Retention and uptake studies

Preparation of coacervate vesicles with sequestered guest components (fluorescent dyes and enzymes) was carried out by following the general procedure described in the methods section, with the guest molecular component added to the PDMAEMA solution before mixing with the polyanion. The concentration of the solutions of guest components was fixed to 1 mg mL^-1^, and the volume added was 5% of the volume of PDMAEMA.

The uptake of the fluorescent dyes inside the vesicles was determined from confocal laser scanning microscopy images by studying the emission line profile across the loaded vesicle. This also allowed for the determination of the equilibrium distribution coefficient, defined as the maximum fluorescence intensity inside the coacervate vesicle membrane and/or aqueous lumen over the averaged fluorescence intensity of the bulk aqueous phase (I_coacervate vesicle_/I_bulk_). The list of all fluorescent dyes used along with the corresponding microscope settings, is provided in **Table S3**. Brightfield images were also acquired to easily identify the membrane of non-fluorescent or low fluorescent coacervate vesicles. All images were analyzed with the last version of ImageJ software.

**Table S3:** Details of the fluorescence confocal laser scanning microscope settings employed in this study.

| **Sample** | **λ_exc_** | **λ_emis_** | **Laser int.** | **Sensitivity** |
| --- | --- | --- | --- | --- |
| Nile Blue | 640 nm | 650 – 750 nm | 2.0 % | 500 V |
| [Ru(bpy)_3_]^2+^ | 488 nm | 600 – 700 nm | 3.0 % | 500 V |
| Rhodamine B | 561 nm | 570 – 670 nm | 0.05 % | 500 V |
| Calcein | 488 nm | 500 – 600 nm | 0.1 % | 470 V |
| Eosin Y | 488 nm | 510 – 610 nm | 0.1 % | 500 V |
| 7-diethylamino-4-methyl coumarin | 375 nm | 420 – 520 nm | 6.5 % | 500 V |
| RITC-tagged GOx | 561 nm | 570 – 670 nm | 2.75 % | 485 V |
| FITC-tagged HRP | 488 nm | 500 – 600 nm | 2.5 % | 485 V |

**Table S4:** Molecular retention an uptake study of coacervate vesicles.


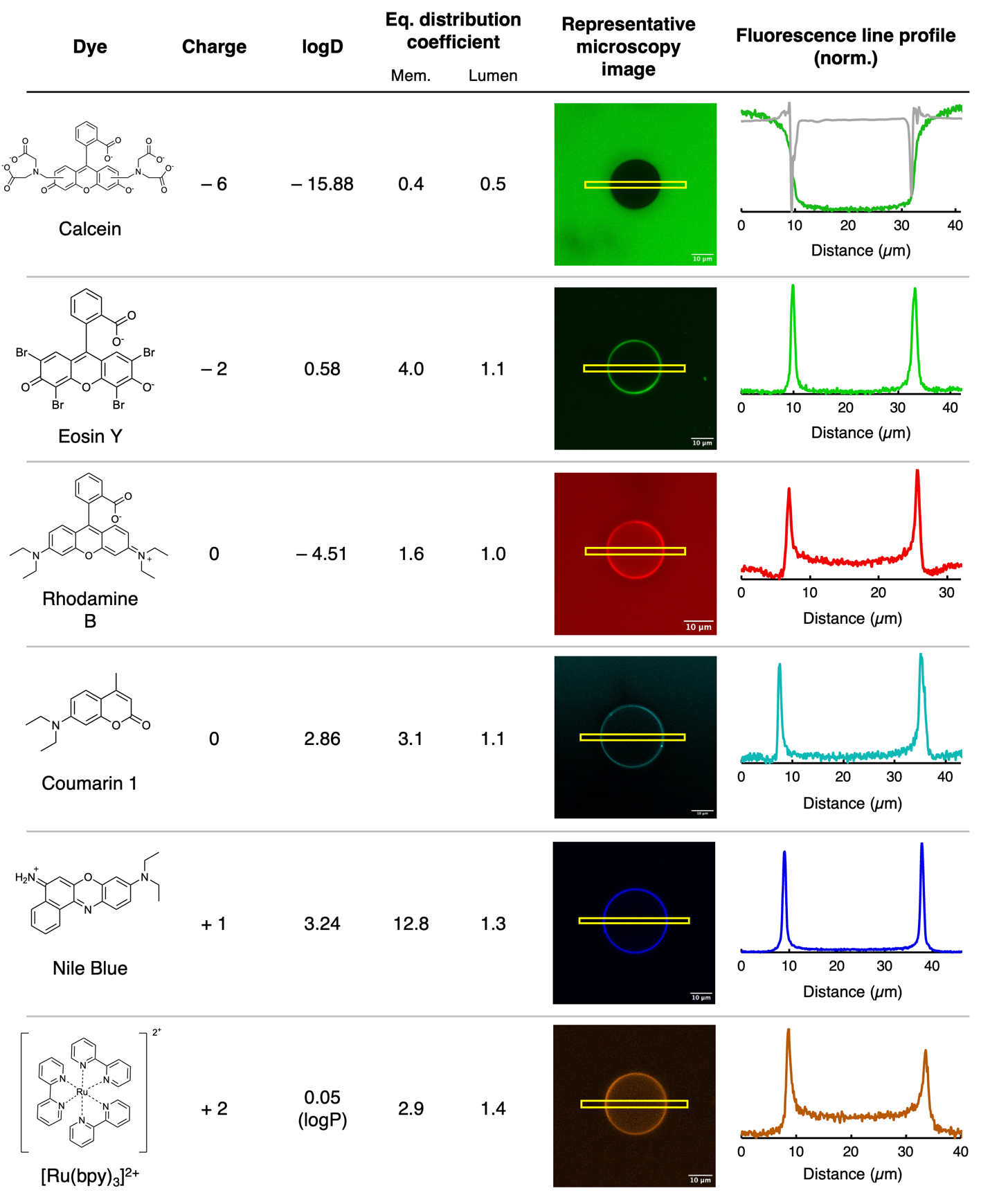


For each molecular dye are reported: (first column) the name and its structure; (second column) net charge at pH 6.5; (third column) water/octanol equilibrium distribution coefficients (logD); (fourth column) equilibrium distribution coefficient referred to the membrane and the internal aqueous lumen, respectively; (fifth column) a representative confocal laser scanning microscopy image; (sixth column) a normalized fluorescence intensity line profile referred to the yellow area on the corresponding microscopy image.

Molecular structures at pH 6.5, water/octanol equilibrium distribution coefficients (logD) and net charges were calculated with MarvinSketch (Chemaxon).

An equilibrium distribution coefficient value greater than 1 indicates that the fluorescent species is sequestered inside the coacervate vesicles membrane or lumen (*e.g.*, Nile Blue, [Ru(bpy)]^2+^, rhodamine B, eosin Y, 7-diethylamino-4-methylcoumarin). On the other hand, an equilibrium distribution coefficient less than 1 indicates that the fluorescent species preferentially distributes in the external solution (*e.g.*, Calcein). The graph of the fluorescence line profile of Calcein presents an additional grey plot that refers to the brightfield intensity line profile in the same yellow area, where the minima of intensity correspond to the membrane of the vesicle.

1. Supplementary discussion of molecular uptake results

Fluorescent dye uptake experiments revealed selective sequestration patterns in coacervate vesicle membranes and lumens, governed by molecular charge at pH 6.5 and hydrophobicity (**Fig. 5a**, **Table S4**). The vesicles' negative charge and hydrophobic nature led to distinct distribution patterns between these two regions.

Membrane sequestration was primarily driven by hydrophobicity, with molecular charge playing a secondary role. For example, Nile Blue, positively charged and highly hydrophobic, was effectively and selectively sequestered in the membrane, whereas [Ru(bpy)_3_]^2+^, being positively charged but weakly hydrophobic, displayed only a discrete presence in the membrane. The Coumarin derivative, neutral and highly hydrophobic, and Eosin Y, negatively charged but hydrophobic, were discretely sequestered in the membrane. Rhodamine B, neutral but hydrophilic, showed weak sequestration in the membrane, and Calcein highly negatively charged and highly hydrophilic exhibited no membrane sequestration and remained in the bulk aqueous solution.

In contrast, lumen distribution was predominantly determined by molecular charge, favoring positively charged, weakly hydrophobic molecules. For example, [Ru(bpy)_3_]^2+^ bearing the highest positive charge and being weakly hydrophobic showed the highest presence in the lumen, followed by Nile Blue, positively charged but highly hydrophobic, the neutral Coumarin 1, and by the negatively charged dyes.

Interestingly, confocal laser scanning microscopy revealed distinct localization patterns when RITC-tagged GOx and FITC-tagged HRP were used in uptake experiments (main text **Fig. 5c,d**, **Table S5**). RITC-tagged GOx predominantly accumulated in the vesicle membrane, while FITC-tagged HRP selectively concentrated within the aqueous lumen. This spatial segregation arose from the enzymes' different zeta potentials, hydropathy, and sizes, which governed their interactions with the self-assembled polyelectrolyte microcompartments. GOx is a large enzyme (Mw 160 kDa, including carbohydrates) with an isoelectric point (pI) between 3.9 and 4.3.^9^ At the working pH of 6.5, GOx is weakly negatively charged, as confirmed by zeta potential measurements (–10.0 ± 0.8 mV; **Table S5**). Computational modelling reveals that its surface is dominated by negatively charged and hydrophobic patches (**Fig. S18**). These properties cause RITC-tagged GOx to behave similarly to Eosin Y, leading to its sequestration in the vesicle membrane with a high distribution coefficient.

In contrast, HRP has a molecular weight of 44 kDa (including carbohydrates) and a pI of 7.2.^10^ At pH 6.5, HRP is weakly positively charged, as supported by zeta potential measurements (+4.5 ± 1.0 mV; **Table S5**). Computational modelling indicates that its surface is primarily composed of positively charged and hydrophilic patches (**Fig. S18**). Consequently, FITC-tagged HRP behaves similarly to [Ru(bpy)_3_]^2+^ and is predominantly sequestered in the vesicle lumen.

The segregation process may also be influenced by enzyme size. The large molecular weight of GOx likely causes it to become kinetically trapped in the membrane during impulsive force-driven self-assembly. Its negative charge may further facilitate nanocoacervates co-penetration and adhesion. In contrast, the smaller size, positive charge, and hydrophilic nature of HRP allow it to be easily displaced from the negatively charged nanocoacervates, leading to its entrapment in the vesicle lumen.

1. Enzyme labelling

In general, 10 mg of enzyme were dissolved in 73.5 mL of Na_2_CO_3_ buffer (pH 8.5, 100 mM). Fluorescent tags fluorescein isothiocyanate (FITC) or rhodamine isothiocyanate (RITC) were dissolved in DMSO to yield 1 mg mL^-1^ stock solutions. 100 μL of the fluorescent tag solution was added to the enzyme aqueous solution and the mixture was stirred overnight at 4 °C. The product was purified by dialysis, lyophilized and stored as a solid at −20 °C.

The degree of labelling of the tagged enzymes was calculated using UV-vis spectroscopy [FITC *ɛ_485 nm_* = 50,400 L mol^-1^ cm^-1^, GOx *ɛ_280 nm_* = 3,006,000 L mol^-1^ cm^-1^, RITC *ɛ_559 nm_* = 62,100 L mol^-1^ cm^-1^, HRP *ɛ_403 nm_* = 102,000 L mol^-1^ cm^-1^] and determined to be 14.6 GOx-RITC and 2.7 for HRP-FITC.

1. In-vitro evolution of a binary population of coacervate vesicles

Enzyme-loaded coacervate vesicles were prepared as described in the methods section. Separate populations of first-generation coacervate vesicles were formed, each encapsulating either RITC-tagged glucose oxidase (RITC-GOx) or FITC-tagged horseradish peroxidase (FITC-HRP). These two coacervate vesicles populations were then mixed in equal volumes (1:1 ratio) within a 1.75 mL glass vial and imaged using confocal laser scanning microscopy. After seven days, allowing for complete disassembly, the vial was re-shaken for 30 s to form a second-generation of coacervate vesicles containing both enzymes within the same microstructure. Confocal laser scanning microscopy images were taken at this stage to confirm co-encapsulation of the two enzymes. All images were analyzed using fluorescence line intensity profile to localize the two differently tagged enzymes within the vesicle structure.


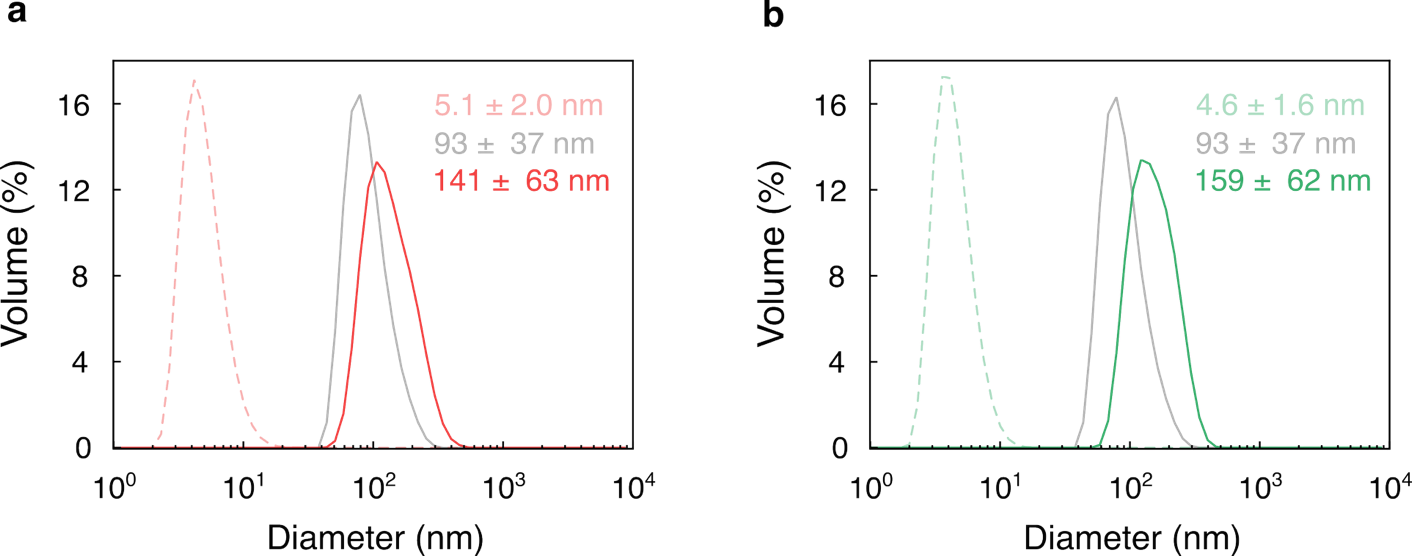


**Fig. S18.** Dynamic light scattering (DLS) analysis of enzyme-loaded nanocoacervates.
**a,** Size distribution profiles comparing: PDMAEMA/PAA (1:4 molar ratio) nanocoacervates (gray plot), the same coacervate system but with encapsulated GOx (red plot), and a 1 mg mL^-1^ GOx solution in H_2_O at pH 6.5 (dashed light red plot).
**b,** Size distribution profiles comparing: PDMAEMA/PAA nanodroplets (gray plot), the same nanodroplets but containing HRP (green plot), and a 1 mg mL^-1^ HRP solution in H_2_O at pH 6.5 (dashed light plot green).

Both systems show an increased nanodroplet diameter upon enzyme engulfment compared to empty coacervates, confirming successful incorporation. The absence of peaks below 10 nm in both (**a**) and (**b**) demonstrates complete enzyme uptake with no detectable free enzyme in solution.

**
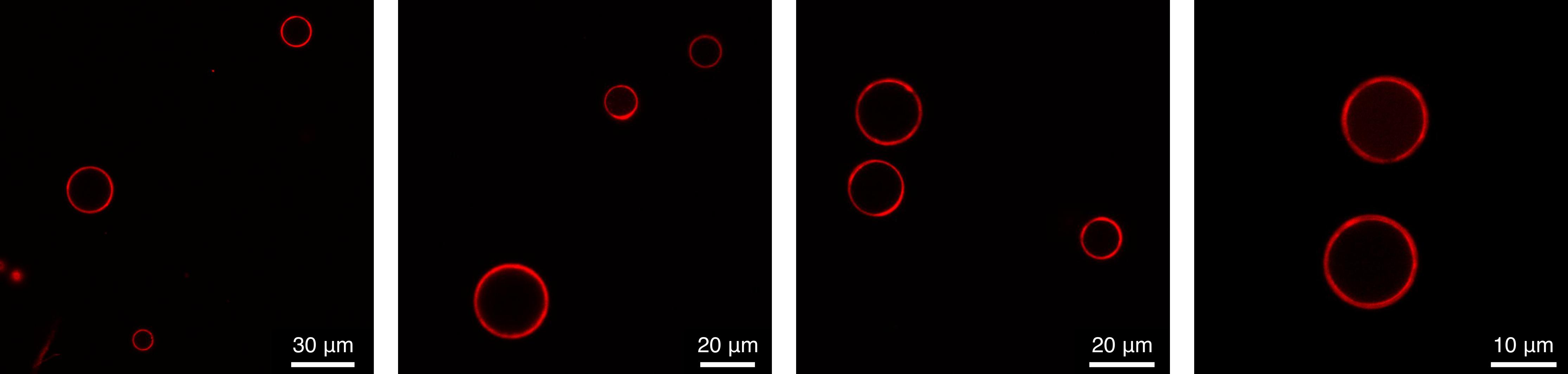
**

**Fig. S19.** Gallery of representative confocal laser scanning microscopy images showing a population of RITC-tagged GOx-loaded coacervate vesicles, prepared as described in the methods section. The red-fluorescent enzyme is clearly localized at the vesicle membrane.


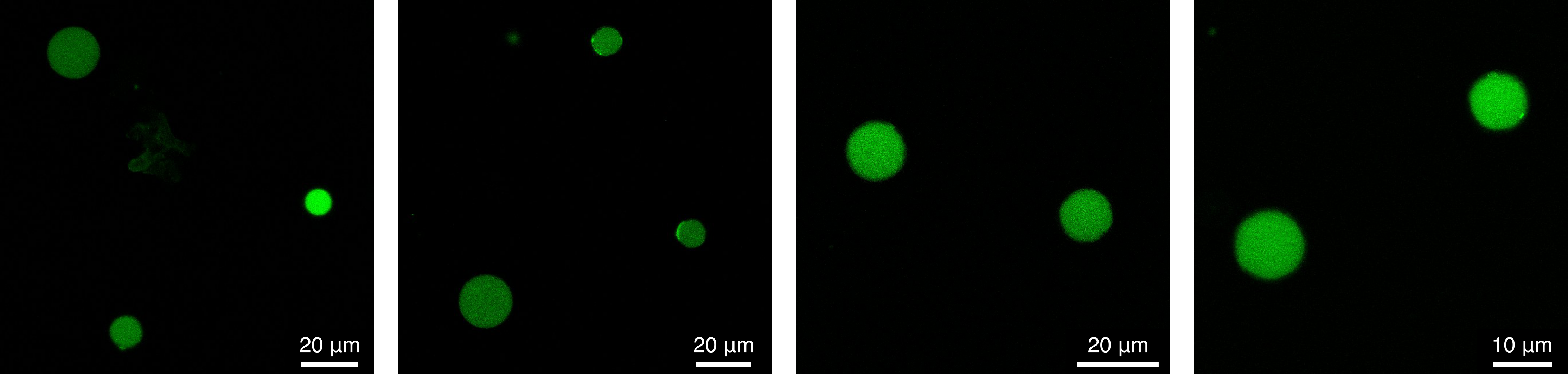


**Fig. S20.** Gallery of representative confocal laser scanning microscopy images showing a population of FITC-tagged HRP-loaded coacervate vesicles, prepared as described in the methods section. In each case the green-fluorescent enzyme is entrapped inside the internal aqueous lumen of the vesicles.

**Table S5:** Enzymatic retention an uptake study of coacervate vesicles.


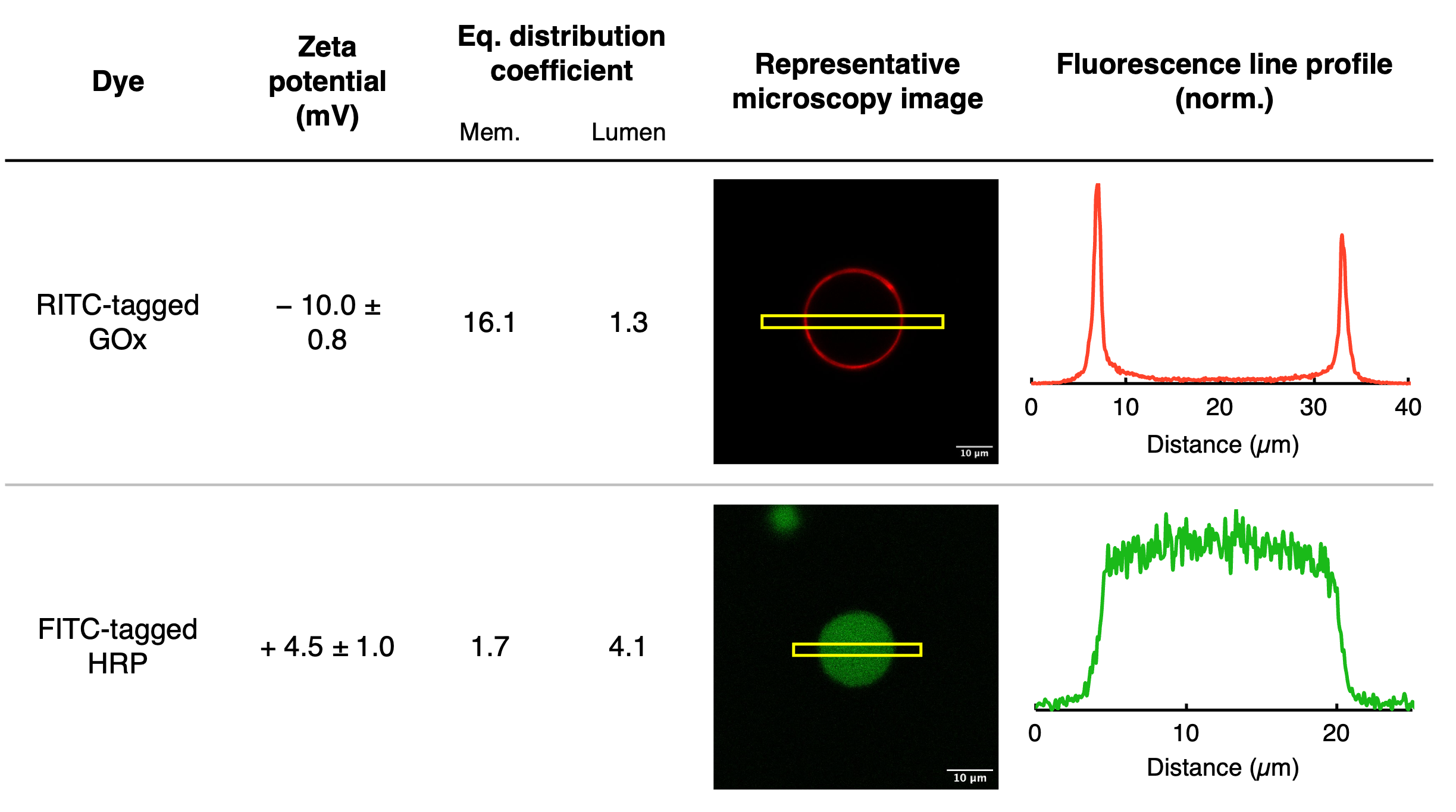


For the two tagged enzymes are reported: (first column) the name; (second column) zeta potential obtained at pH 6.5; (third column) equilibrium distribution coefficient referred to the membrane and the internal aqueous lumen, respectively; (fifth column) a representative confocal laser scanning microscopy image; (sixth column) a normalized fluorescence intensity line profile referred to the yellow area on the corresponding microscopy image.


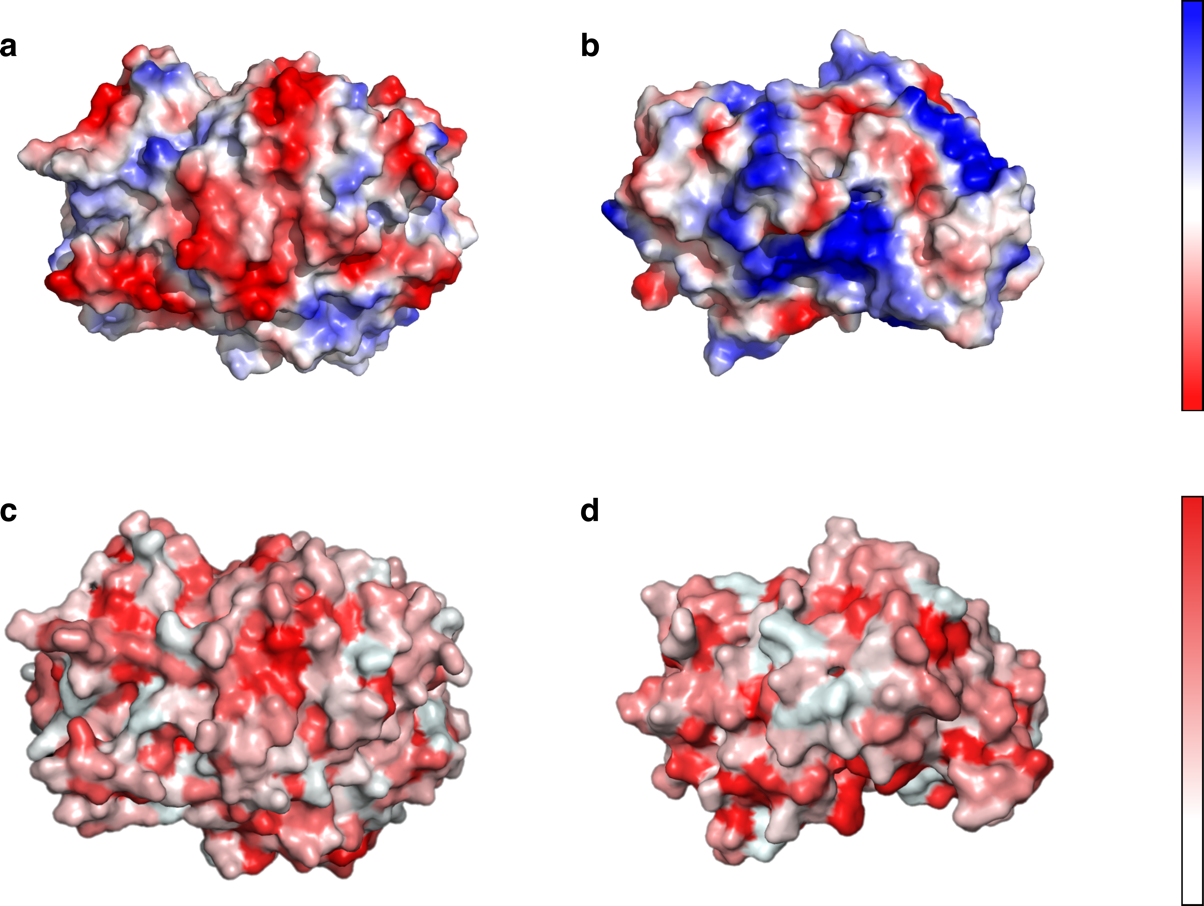


**Fig. S21.** Computational 3D models of GOx and HRP generated with PyMOL. **a,** Charge-smoothed surface representation of GOx, displaying protein contact potentials ranging from
–72.902 eV (red, negative) to 72.902 eV (blue, positive). **b,** Charge-smoothed surface representation of HRP, with potentials ranging from –48.749 eV (red, negative) to 48.749 eV (blue, positive). **c-d,** Surface hydropathy representation of GOx and HRP, respectively. Hydropathy was visualized using the *color_h.py* script (University of Osaka, <https://ouchidekaiseki.com/en/hydrophobicity.php>) based on the Eisenberg scale, where 1.38 (red) indicates the most hydrophobic regions and –2.53 (white) indicates the most hydrophilic regions.^11^

**Table S6:** Retention and uptake study of FITC-tagged dextran into coacervate vesicles.


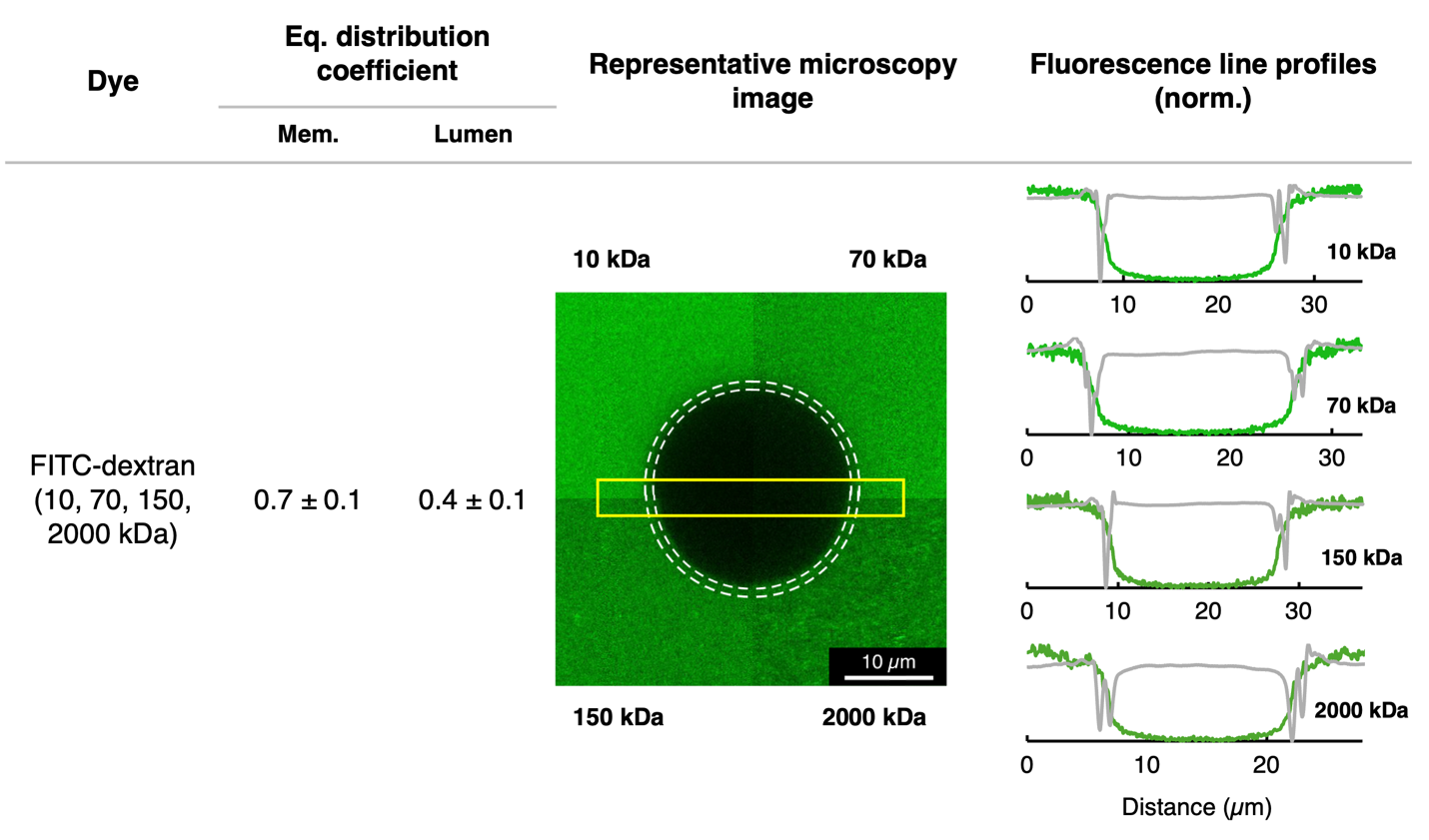


Uptake characterization of FITC-labeled dextran (10, 70, 150, and 2000 kDa) in coacervate vesicles. The columns show: (first) molecular weight; (second) average equilibrium distribution coefficients for the membrane and internal aqueous lumen; (third) combined confocal laser scanning microscopy images of coacervate vesicles prepared in the presence of each dextran molecular weight; (fourth) normalized fluorescence intensity line profiles corresponding to the yellow regions in the microscopy images. The data show that dextran does not enter the vesicle lumen.


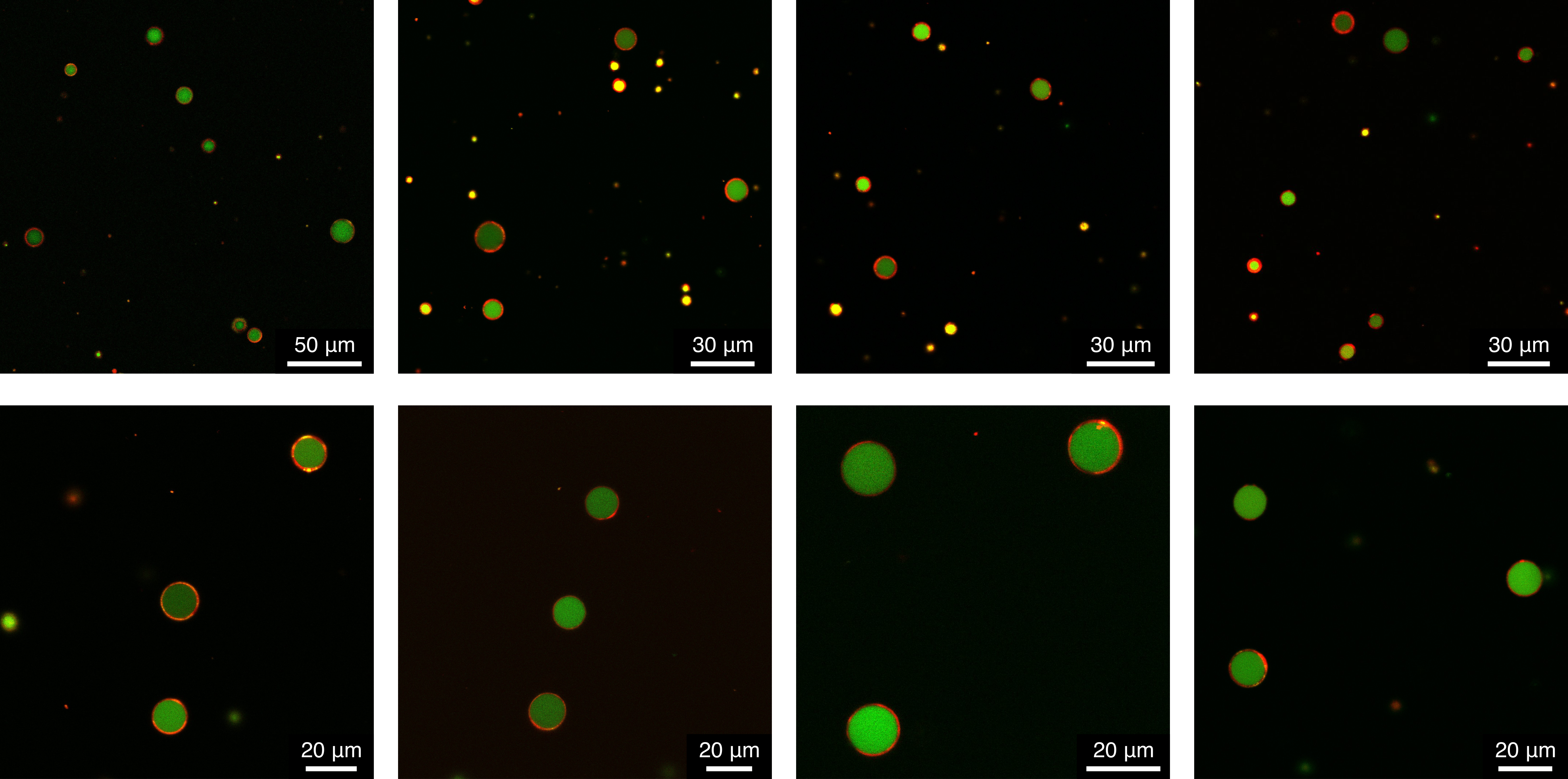


**Fig. S22.** Gallery of representative confocal laser scanning microscopy images showing a population of coacervate vesicles containing both RITC-tagged GOx and FITC-tagged HRP. Coacervate vesicles were prepared as described in the methods section by applying impulsive force to an aqueous solution containing both polymers (PDMAEMA and PAA) along with both GOx and HRP tagged enzymes. In these images, the red-fluorescent GOx (RITC-GOx) is predominantly localized within the vesicle membrane, whereas the green-fluorescent HRP (FITC-HRP) is preferentially confined to the inner aqueous lumen of the vesicles.

1. Hydrogel preparation and enzyme cascade reaction kinetic studies

Due to the gradual sedimentation of the coacervate vesicles over time, fluorescence spectroscopy kinetics for enzyme cascade reactions were performed with the vesicles embedded on an agarose hydrogel matrix, which was immobilized on the side of a 1 mL cuvette. For this, first, a 1 wt% agarose solution was prepared by dissolving agarose powder in Milli-Q water. The mixture was heated in a glass vial at 80°C for 2 hrs, until the agarose was fully dissolved, and the solution became transparent. The agarose solution was then allowed to cool down to 50°C. For the enzyme cascade reaction experiment, 200 µL of the 50 °C agarose solution was immediately mixed with 100 µL of an aqueous suspension of coacervate vesicles (prepared from PDMAEMA 10 mM, 100 µL; non-tagged enzymes solution 1 mg mL^-1^, 5 µL; PAA 10 mM, 400 µL; prepared as mentioned in the methods section) in an Eppendorf tube. The resulting mixture was gently homogenized by pipetting and transferred into a 1 mL Hellma quartz cuvette. The hydrogel was allowed to solidify at 25°C for 15 minutes, resulting in a final hydrogel concentration of approximately 0.66 wt%.

The enzyme cascade reactions were started by adding 400 µL of Milli-Q water to the hydrogel-containing cuvette, followed by the immediate addition of a 15 µL substrate solution (glucose 100 mM, *o*-PD 50 mM, mixed in a 1:1 volume ratio).

The time-dependent enzyme-mediated cascade synthesis of fluorescent 2,3-DAP was monitored for 60 min by fluorescence spectroscopy (*λ_exc_* = 405 nm, *λ_em_* = 550–625 nm, 1 spectrum per minute).


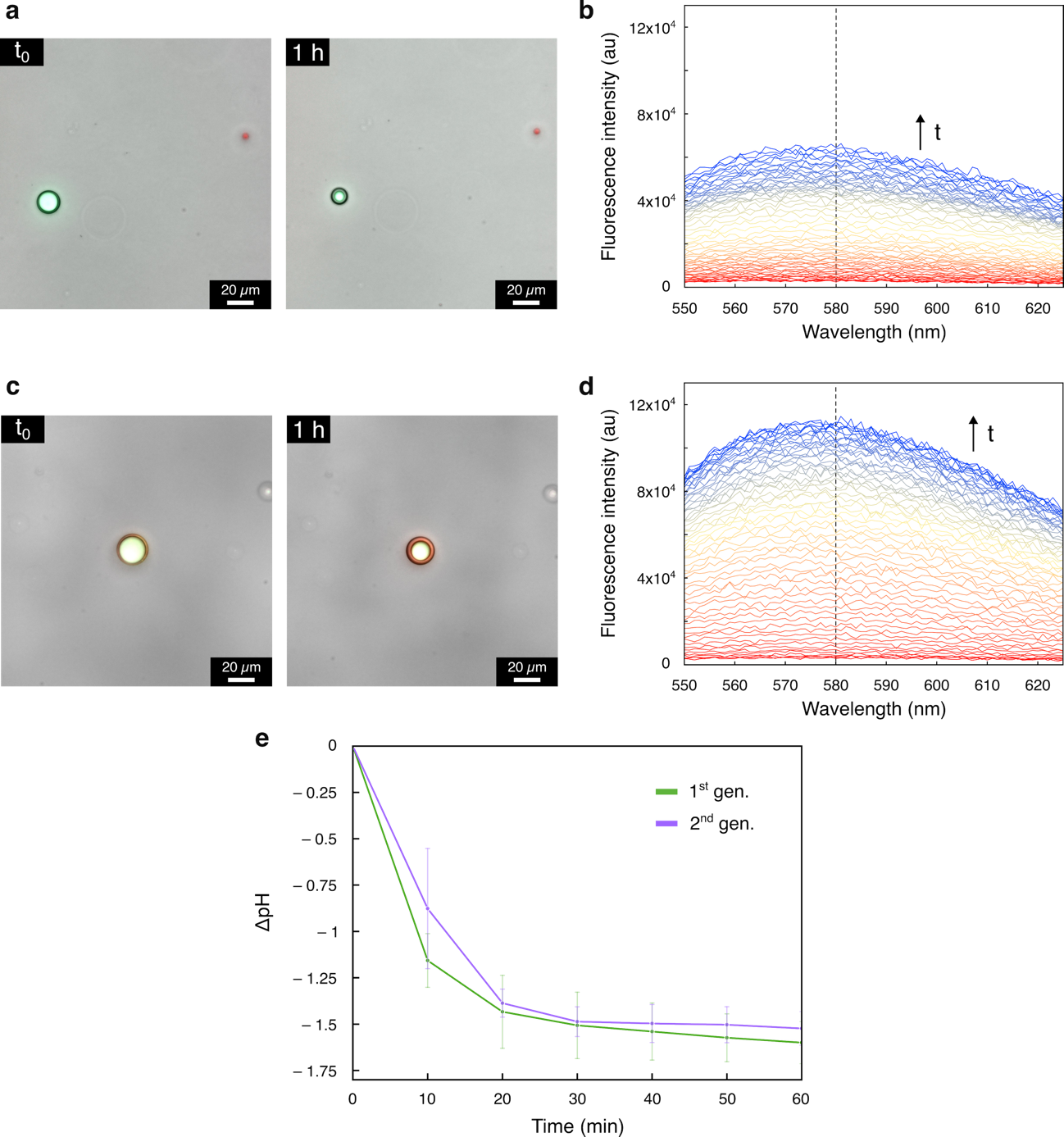


**Fig. S23.** Hydrogel-embedded enzyme-loaded coacervate vesicles and cascade reaction kinetics. **a,** Representative optical microscopy images with overlayed brightfield, red and green fluorescence channels showing a mixed binary population of coacervate vesicles containing only RITC-tagged GOx or FITC-tagged HRP (1^st^ generation) and embedded in an agarose hydrogel matrix. The image on the left was captured immediately after vesicle formation, the image on the right was acquired on the same position after 1 hr. The image after 1 hr shows that within the hydrogel the vesicles behave like in the bulk solution, with their dimeter gradually decreasing overtime and without detectable enzyme leakage. **b,** Kinetic emission spectra of the 1^st^ generation coacervate vesicles following the addition of substrates (glucose and o-PD). The formation of 2,3-DAP was monitored by tracking the emission peak at 580 nm, providing insights into the cascade reaction rate under segregated conditions. **c,** Optical microscopy images (brightfield, red and green channels overlapped) of coacervate vesicles containing both RITC-tagged GOx and FITC-tagged HRP (2^nd^ generation) embedded within an agarose hydrogel matrix. The image on the left was captured immediately after vesicle formation, the image on the right was acquired on the same position after 1 hr. The enzymes display selective localization, with GOx predominantly at the vesicle membrane and HRP within the internal lumen, similarly to what observed in aqueous conditions. **d,** Kinetic emission spectra for the 2^nd^ generation coacervate vesicles, showing faster 2,3-DAP production due to enzyme co-encapsulation within the same vesicle, which facilitates faster reaction kinetics due to the reduced diffusion distance between catalytic sites. Experiments were performed in triplicate, with results summarized in **Fig. 5f**. **e,** Time-dependent pH variations during enzymatic cascade reaction in 1^st^ generation (green plot) and 2^nd^ generation (purple plot) coacervate vesicles. The decrease in pH is associated with the production of gluconic acid following the hydrolysis of gluconolactone. The comparable kinetics between the two systems confirms similar GOx activity, demonstrating that the enhanced overall reaction rate of 2,3-DAP production in the 2^nd^-generation system arises specifically from improved H_2_O_2_ diffusion rather than differences in the first catalytic step. Error bands represent standard deviation (n = 3).

**References**

1. Chapman, R., Gormley, A. J., Stenzel, M. H. & Stevens, M. M. Combinatorial Low‐Volume Synthesis of Well‐Defined Polymers by Enzyme Degassing. *Angew. Chem. Int. Ed.* **55**, 4500–4503 (2016).

2. Glasoe, P. K. & Long, F. A. USE OF GLASS ELECTRODES TO MEASURE ACIDITIES IN DEUTERIUM OXIDE^1,2^. *J. Phys. Chem.* **64**, 188–190 (1960).

3. Spruijt, E. *et al.* Structure and Dynamics of Polyelectrolyte Complex Coacervates Studied by Scattering of Neutrons, X-rays, and Light. *Macromolecules* **46**, 4596–4605 (2013).

4. Dunlop, I. E. *et al.* Direct Measurement of Normal and Shear Forces between Surface-Grown Polyelectrolyte Layers. *J. Phys. Chem. B* **113**, 3947–3956 (2009).

5. Borkovec, M. *et al.* Investigating forces between charged particles in the presence of oppositely charged polyelectrolytes with the multi-particle colloidal probe technique. *Adv. Colloid Interface Sci.* **179–182**, 85–98 (2012).

6. De Gennes, P. G. Polymers at an interface; a simplified view. *Adv. Colloid Interface Sci.* **27**, 189–209 (1987).

7. Soto, D., De Larivière, A. B., Boutillon, X., Clanet, C. & Quéré, D. The force of impacting rain. *Soft Matter* **10**, 4929–4934 (2014).

8. Blackmore, P. A. & Hewson, P. J. Experiments on full-scale wave impact pressures. *Coast. Eng.* **8**, 331–346 (1984).

9. Wilson, R. & Turner, A. P. F. Glucose oxidase: an ideal enzyme. *Biosens. Bioelectron.* **7**, 165–185 (1992).

10. Maehly, A. C. [143] Plant peroxidase. in *Methods in Enzymology* vol. 2 801–813 (Elsevier, 1955).

11. Eisenberg, D., Schwarz, E., Komaromy, M. & Wall, R. Analysis of membrane and surface protein sequences with the hydrophobic moment plot. *J. Mol. Biol.* **179**, 125–142 (1984).
